# Transient Propagation of the Invasion Front in the Homogeneous Landscape and in the Presence of a Road

**DOI:** 10.1007/s11538-024-01302-3

**Published:** 2024-05-22

**Authors:** Bradly Deeley, Natalia Petrovskaya

**Affiliations:** School of Mathematics, University of Birmingham, Birmingham, UK

**Keywords:** Invasive plants, Spread rate, Fragmented landscape, Travelling wave, Transient regime, Integro-difference equation

## Abstract

Understanding the propagation of invasive plants at the beginning of invasive spread is important as it can help practitioners eradicate harmful species more efficiently. In our work the propagation regime of the invasive plant species is studied at the short-time scale before a travelling wave is established and advances into space at a constant speed. The integro-difference framework has been employed to deal with a stage-structured population, and a short-distance dispersal mode has been considered in the homogeneous environment and when a road presents in the landscape. It is explained in the paper how nonlinear spatio-temporal dynamics arise in a transient regime where the propagation speed depends on the detection threshold population density. Furthermore, we investigate the question of whether the transient dynamics become different when the homogeneous landscape is transformed into the heterogeneous one. It is shown in the paper how invasion slows down in a transient regime in the presence of a road.

## Introduction

Invasive plant species in various habitats around the world cause significant economic and environmental damage (Cuthbert et al. 2021). In order to effectively control, manage, and predict the future distribution of invasive plant species, it is essential to accurately understand the propagation speed of the invasive species in different landscapes and at all stages of invasion. The problem of how organisms spread in the spatial domain has long been a topic of interest (Andow et al. 1990), and the first key results pertaining to the progression of invasion into the spatial domain were achieved in Fisher (1937), where the existence of a travelling wave was demonstrated when a mutation propagates in the homogeneous environment. Convergence of the initial condition to the travelling wave solution has been proved in Kolmogorov et al. (1937), and further generalisation of the results has been provided in Weinberger (1982). The propagation speed of a wave of advancement in the homogeneous landscape has been investigated both analytically and numerically for a range of dispersal kernels and growth functions (Hadeler and Rothe 1975; Kot et al. 1996; Li and Otto 2022; Sherratt and Marchant 1996; Wang et al. 2002). There have also been a number of studies that have demonstrated how to evaluate the propagation speed from data (Lewis et al. 2006) and that have used field data to model the propagation of a specific species of flora (Caplat et al. 2012; Lehsten et al. 2019). Much work has also been carried out previously investigating the propagation speed in systems with more complex behaviour, for example, in predator–prey dynamics (Lewis et al. 2016; Rodrigues et al. 2015; Wang et al. 2021), or including mutations (Lutscher et al. 2022) in the homogeneous environment. The propagation in heterogeneous landscapes has also been investigated (Lewis et al. 2016; Lutscher 2019; Shigesada and Kawasaki 1997).

Most of the studies on the propagation of invasive species have focused on the later spread once a travelling wave of invasion has become established in the domain, and short-time behaviour before the propagation speed follows the long-time established behaviour has not received a lot of attention. In the homogeneous case, it has been shown in Petrovskii and Shigesada (2001) that the population density can display non-monotonic behaviour at the short-time interval due to the choice of the initial condition, yet little detail has been given about propagation regimes at the beginning of the invasive spread. Meanwhile, the benefits of preventing the invasive spread are well-known (Leung et al. 2002; Weber and Gut 2004) and the importance of eradicating invasive plants at the early stage of invasion when plants that have naturalised start spreading is recognised (Andow et al. 2003; Blood et al. 2019). Hence, it is important to understand the propagation of an invasive plant at the early stages of the invasive spread. Furthermore, in the case when the asymptotic propagation speed does not change with time, a transient regime contributes to the overall propagation of the invasive species. Depending on the landscape features in the spatial domain, a shorter or longer distance may be covered by the invasive species in a transient regime and the difference in the size of the invaded domain will not disappear at the long-time scale.

In our paper, we consider propagation of invasive plant species in the homogeneous and heterogeneous environment. A particular focus in our study is on the initial short-time period of the spread before a monotone travelling wave of the population density has been established to advance into space at a constant speed. It is well-known (e.g., see Lutscher 2019) that the propagation speed in a transient regime depends on the minimum population density at which the invasive species is detected. However, the definition of the detection threshold density is heavily related to the detection and monitoring method and sensitivity used in monitoring protocols (e.g., see Haber 1997; Luo et al. 2022; Shiri et al. 2023). The question of to what extent the transient dynamics are changed when the detection threshold density is varied has to be investigated and we first provide the study of this question in the homogeneous environment before moving to a more challenging case of the heterogeneous landscape.

Various heterogeneous disturbances to the homogeneous landscape may result in different spatio-temporal dynamics in comparison with the ‘baseline’ homogeneous case. Hence, the question of whether the transient dynamics become different when the homogeneous landscape is transformed into the heterogeneous one requires specification of the heterogeneity in the problem. In our paper we investigate this question when heterogeneity is introduced as a road in the spatial domain. Our choice of the study case follows the previous work (Deeley and Petrovskaya 2022) where a model of propagation of the invasive species at the presence of a road was introduced. Roads are an increasingly essential part of the human-modified landscape and it is estimated that there will be millions of additional kilometers of roads by 2050 (Dulac 2013; Meijer et al. 2018). Consequently, roads can cause a large number of detrimental environmental effects (Ibisch 2016) and impact the movement of invasive plant species (Deeley and Petrovskaya 2022). It is, therefore, essential to investigate how the propagation of invasive plants change when the homogeneous landscape is modified by constructing a road.

The paper is organised as follows. In the next section, we recall a mathematical model of biological invasion introduced originally in Deeley and Petrovskaya (2022). The integro-difference framework is employed in the model to simulate spatio-temporal dynamics of a stage-structured invasive plant in the homogeneous and heterogeneous landscape. In Sect. 3, we study the propagation of the invasive plant species in a transient regime in the homogeneous environment.We will argue that the propagation speed is sensitive to the choice of the detection threshold density alone when the other parameters, including the definition of the initial condition, are fixed in the problem. We then perform a similar study in Sect. 4 to understand how the invasive plant propagates at the short-time scale in the heterogeneous environment. We will demonstrate in Sect. 4 how the propagation of the invasive species at short times is changed by the presence of a road in the model. Among other parameters, we are interested in the impact the road width makes on the propagation regime at short times, and it will be argued in Sect. 4 that invasion slows down in the presence of a wide road. Concluding remarks are then provided in Sect. 5, where we also discuss directions of future work.

## The Model

A generic model of plants propagating in the landscape with a road was formulated in Deeley and Petrovskaya (2022), and here we re-introduce it briefly for the sake of further discussion. An invasive plant species is described in the model by its population density *N*, where the life cycle of the species exhibits two distinctly different stages: the demographic stage which can include the growth of juveniles, their maturation and reproduction (e.g., on a yearly cycle through seed dispersal or pollination (Lewis et al. 2016)), followed by the dispersal stage. Hence, we use a discrete-time framework (Kot and Schaffer 1986) where the population density evolves from generation *t* to generation $t+1$, i.e., we consider the discrete time with the increment $\delta t=1$.

Let $N(t,x) \equiv N_t(x)$ be the population density in generation *t* over continuous space *x*. We consider the propagation-growth model in dimensionless form. The demographic stage is described as

1 $$\begin{aligned} {{N}^{gr}_{t}}(x)=F(N_t(x)), \end{aligned}$$

where $N_t(x)$ is the species’ spatial distribution emerging after the dispersal stage in the previous generation, and $F(N_t)$ is the growth function whose definition will be discussed later in the text. After the demographic stage of the given generation is complete, the species enter the dispersal stage, which, when finished, produces the species’ spatial distribution in the next generation:

2 $$\begin{aligned} N_{t+1}(x)=\int _{\Omega }{{N}^{gr}_{t}}(y)k(x,y)dy, \end{aligned}$$

where $\Omega$ is a dispersal domain and *k*(*x*, *y*) is a dispersal kernel. Our present study is restricted by a simple case of the Gaussian dispersal kernel given by

3 $$\begin{aligned} k({x},y)=\frac{1}{\sqrt{2\pi \sigma ^2}}\exp \left( -\frac{(x-y)^2}{2\sigma ^2}\right) . \end{aligned}$$

The standard deviation $\sigma$ in (3) is the parameter quantifying the spatial scale of the dispersal. Having substituted (1) into (2), we obtain the following integro-difference equation for the population density in generation $t+1$ in the spatial domain $\Omega$:

4 $$\begin{aligned} {N}_{t+1}(x) = \int _\Omega F\left( N_t(y)\right) k(x,y)\,dy. \end{aligned}$$

The equation (4) is solved along with the definition of the growth function *F*(*N*) and relevant initial condition in the spatial domain $\Omega$.

We now include a ‘road’ subdomain $\Omega _R \subset \Omega$ into the model, where we account for heterogeneity present in the landscape by introducing an additional dispersal stage. The time interval from *t* to $t+1$ is split into two sub-intervals $[t, t+\frac{1}{2}]$ and $[t+\frac{1}{2}, t+1]$. First, the solution in the entire domain $\Omega$ is obtained, where we now consider the Eq. (4) at time $\displaystyle {t+\frac{1}{2}}$. Next, we identify the population density $N^R$ for the region of the road as follows

5 $$\begin{aligned} N^R_{t+\frac{1}{2}}(x) = {\left\{ \begin{array}{ll} N_{t+\frac{1}{2}}(x), &{} \text {if } x\in \Omega _R,\\ 0, &{} \text {otherwise,} \end{array}\right. } \end{aligned}$$

and we apply an additional dispersal step to the road under the assumption that the population is taken away from the road quickly (e.g., by the wind). We have

6 $$\begin{aligned} N^R_{t+1}(x) = \int _{\Omega }N^R_{t+\frac{1}{2}}(y) k(x,y)\, dy. \end{aligned}$$

The additional dispersal (6) moves the population away from the road and contributes to the population density distribution at time $t+1$ as follows

7 $$\begin{aligned} \hat{N}_{t+1}(x) = N_{t+\frac{1}{2}}(x) + N^R_{t+1}(x), \,\,x\in \Omega . \end{aligned}$$

Finally, we assume that the population cannot grow on the road and dies out, i.e., we have

8 $$\begin{aligned} N_{t+1}(x) = {\left\{ \begin{array}{ll} 0 , &{} \text {if } x\in \Omega _R, \\ \hat{N}_{t+1}(x) , &{} \text {otherwise,} \end{array}\right. } \end{aligned}$$

obtaining the population density distribution over the domain $\Omega$ at time $t+1$.

The generic model (4)–(8) requires a definition of the growth function *F*(*N*) and in our study we use the Ricker growth function (Ricker 1954),

9 $$\begin{aligned} F(N) = AN\exp (-N), \, A>1. \end{aligned}$$

The growth model (9) originally developed to predict population growth in fisheries has also been used in plant-focused studies (Andersen 1991; Mayfield and Stouffer 2017; Stouffer 2022) where one advantage of the growth function (9) considered by biologists is that it can never predict negative fecundities in plants (Stouffer 2022).

The steady states $\bar{N}$ of a non-spatial problem that corresponds to the demographic stage (1), (9) are $\bar{N_1}=0$ and $\bar{N_2}=\ln (A)$, where the non-trivial steady state is stable for $0<\ln (A)<2$. Although the growth function (9) presents a wealth of spatio-temporal dynamics regimes for various values of the growth parameter $\ln {(A)}$, (Andersen 1991; Bourgeois et al. 2020; Kot and Schaffer 1986), here we restrict our consideration by the simplest case $0<\ln (A)<1$ providing monotone solutions converging to a travelling wave of the constant height $\ln {(A)}$ (Lutscher 2019). Furthermore, in our model, the growth function (9) can be approximated by a linear growth function with the same growth factor *A*,

10 $$\begin{aligned} F(N) = AN, \end{aligned}$$

as we assume that the initial population density is low and there is no significant increase in the population size at the beginning of invasion.

The population density at time $t=0$ is selected as a Gaussian distribution with mean $\mu =0$, standard deviation $\sigma _0$, and total population density $\Phi$,

11 $$\begin{aligned} N_0(x) = \frac{\Phi }{\sqrt{2\pi \sigma ^2_0}} \exp {\left( -\frac{x^2}{2\sigma ^2_0}\right) }. \end{aligned}$$

Given the growth function and the initial condition, the model (4)–(8) is solved for $t>0$ in the spatial domain $\Omega$. We refer the interested reader to (Deeley and Petrovskaya 2022) for further details of the analytical and numerical solution of the model.

## Invasion at the Short-Time Scale in the Homogeneous Environment

**Fig. 1:**
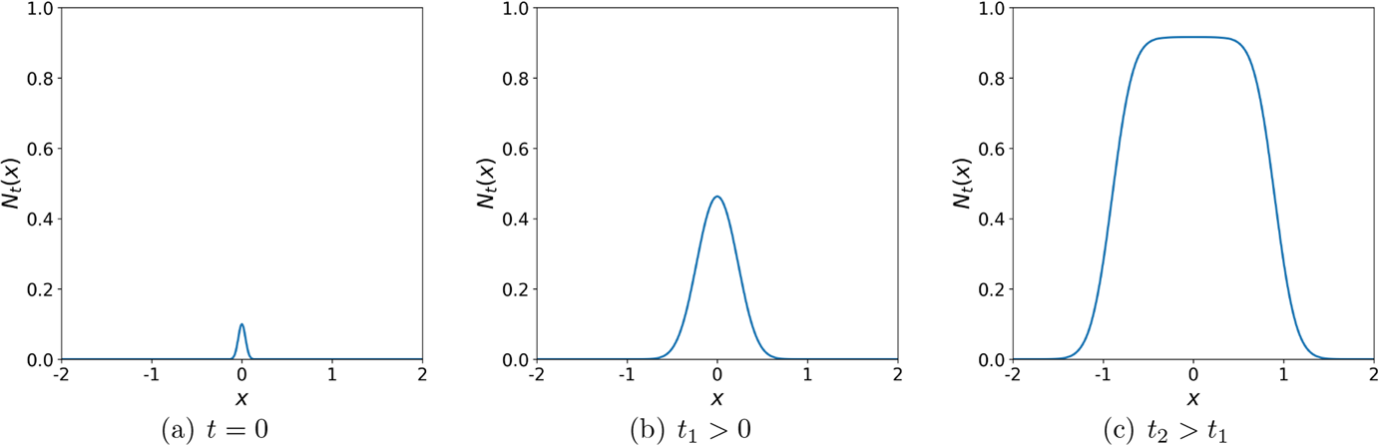
The propagation of the invasive species in the no road case. The parameters used in computation are $\Phi = 0.01$, $h =0.1$, $\sigma = 0.1$, and $A=2.5$. **a** The initial population density at $t=0$ is given by (11). **b** After some time $t_1>0$ the population density grows and spreads further into the spatial domain. **c** At a later time $t_2>t_1$ the population density continues to grow and spread into the domain. The propagating wave will reach the carrying capacity defined by the growth function (9) and will spread at a constant speed as the time progresses (cf. Fig. 7a where a propagating front has already been established in a spatial domain behind the road)

We first set the width $\delta =0$ of the road sub-domain $\Omega _R$ where we refer to this problem as the ‘no road’ case. An example of the solution of the problem (4) with the growth function (9) is shown in Fig. 1 where the initial condition (11) evolves gradually into a travelling wave of the constant height $\ln {(A)}$. We aim to investigate the propagation of the invasive species at the short-time scale before the travelling wave is established (see Fig. 1b), as that will further allow us to analyse the early stage of spread in the heterogeneous ‘road’ landscape.

Given the choice of the dispersal kernel (3), consideration of the linear growth function (10) in the model allows for exact solution. The no road model is defined by the equation

$$\begin{aligned} N_t(x) = \int _{\Omega }\frac{AN_{t-1}(y)}{\sqrt{2\pi \sigma ^2}} \exp {\left( -\frac{(x-y)^2}{2\sigma ^2}\right) } dy, \end{aligned}$$

complemented by the initial condition (11) and solved everywhere in $\Omega$. Since a convolution of a Gaussian dispersal kernel with the initial condition (11) is itself a Gaussian function (e.g., see Lutscher 2019), the exact solution for $t>0$ is given by

12 $$\begin{aligned} N_t(x) = \frac{\Phi A^t}{\sqrt{2\pi (\sigma ^2_0+t\sigma ^2)}} \exp {\left( -\frac{x^2}{2(\sigma ^2_0 + t\sigma ^2)}\right) }. \end{aligned}$$

Let us now assume that the invasive species is monitored in the spatial domain and, as a result of the monitoring, the population density $\tilde{N}$ is detected at the spatial location $x_t>0$ at the time *t*. By rearranging (12) for $x_t(\tilde{N})$ one finds,

13 $$\begin{aligned} x_t(\tilde{N}) = \sqrt{2(\sigma ^2_0+t\sigma ^2) \ln {\left( \frac{\Phi A^t}{\tilde{N} \sqrt{2\pi \left( \sigma ^2_0+t\sigma ^2\right) }}\right) }}. \end{aligned}$$

As the invasive species propagates, the same population density $\tilde{N}$ will be observed in the monitoring routine at a different location. The propagation speed $c=\displaystyle {\frac{\delta x}{\delta t}}$ can be calculated for $\delta t=1$ as (*e.g.*, see Lutscher 2019)

14 $$\begin{aligned} c(t, \tilde{N}) =x_{t}(\tilde{N}) - x_{t-1}(\tilde{N}), \end{aligned}$$

where $x_t$ and $x_{t-1}$ are defined from (13) for any $t>0$. We will refer to the population density $\tilde{N}$ in (14) as *the detection threshold*.

It has been proved in Weinberger (1982) (see also Thieme 1979) that, under certain conditions imposed on a dispersal kernel and a growth function in (4), there is a constant asymptotic spreading speed ${c}^*$, i.e., after an initial transition period the long-time behaviour ${c}^*$ of the propagation speed $c(t, \tilde{N})$ does not depend on the choice of the initial condition and the detection threshold. For the growth function (9) and the dispersal kernel (3), the asymptotic spreading speed is given by (see e.g., Lewis et al. 2016; Lutscher 2019)

15 $$\begin{aligned} {c}^* = \sqrt{2\sigma ^2\ln {(A)}}. \end{aligned}$$

Furthermore, under the modelling choices and assumptions presented about the dispersal kernel and the growth function in Sect. 2, the asymptotic spreading speed is linearly determinate, i.e., the asymptotic speed (15) is the same when the model is linearized and (9) is replaced by the linear growth function (10) (Castillo-Chavez et al. 2013; van den Bosch et al. 1990; Weinberger et al. 2002).

The choice of a detection threshold in (14) requires careful consideration: when the propagation speed is evaluated from (14) in the asymptotic regime (15) the threshold value should be considered as low as possible to provide an accurate linear approximation (10) of the growth function (9). In the direct computation of the propagation speed, the above requirement can be achieved by setting ${\tilde{N}} = \alpha \,\ln {(A)}$, where $\alpha \in \mathbb {R}$ and $\alpha \ll 1$.

Meanwhile, the definition of a ‘low threshold’ is not clear in a transient regime when a travelling wave has not approached the carrying capacity yet. It can be convenient to relate the threshold density ${\tilde{N}}$ to the population size at time $t=0$. Namely, the threshold value is now chosen as ${\tilde{N}}=\alpha h$, where the height $h=N_0(0)$ of the Gaussian ‘hump’ is defined by the parameters $\Phi$ and $\sigma _0$ in (11),

16 $$\begin{aligned} h=\frac{\Phi }{\sigma _0\sqrt{2\pi }}. \end{aligned}$$

If accurate information about the initial population distribution (11) is available, then the requirement of a low threshold holds for $\alpha \ll 1$. However, it has been demonstrated in Petrovskaya and Embleton (2013) that highly aggregated spatial distributions appearing at early stages of biological invasion may be detected with very low accuracy resulting in uncertainty when the total population size is evaluated. Furthermore, the detection threshold density is heavily related to the detection and monitoring method and sensitivity used in monitoring protocols, e.g., see (Haber 1997; Luo et al. 2022; Shiri et al. 2023), and it is not always clear how even a slight change in the threshold density may affect conclusions about propagation of the invasive species before a traveling wave is established at the carrying capacity. Hence in our study, we consider the entire range $0<{\tilde{N}} \le \ln {(A)}$ of the detection threshold to investigate the above question.

**Fig. 2:**
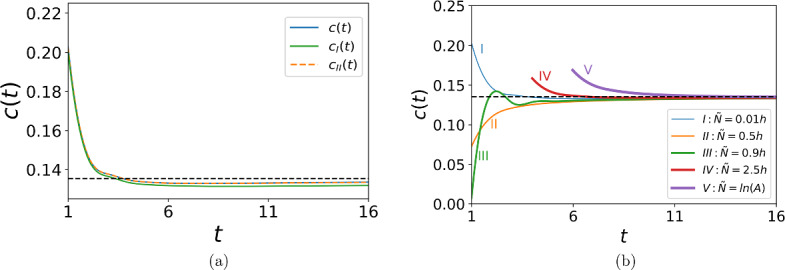
The propagation speed *c*(*t*). The function *c*(*t*) converges to the asymptotic propagation speed (15) (black dashed line in the figure), yet its behaviour at the short-time scale depends on the detection threshold. **a** Comparison between the propagation speed $c_{I}(t)$ obtained by direct computation for the growth function (9) (green solid line), the propagation speed $c_{II}(t)$ obtained by direct computation for the linear growth function (10) (orange dashed line), and the propagation speed *c*(*t*) obtained from (13)–(14) (blue solid line). The parameters are $h = 0.1$, $\Phi = 0.01$, $\tilde{N} = 0.01h$, $A=2.5$, and $\sigma =0.1$. **b** The propagation speed *c*(*t*) (13)–(14) for various choices of the detection threshold: $\tilde{N} = 0.01 h$ (blue solid line), $\tilde{N} = 0.5 h$ (orange solid line), $\tilde{N} = 0.9 h$ (green solid line), $\tilde{N} = 2.5h$ (red solid line), $\tilde{N} = \ln {(A)}$ (purple solid line). The other parameters are the same as in **a**. For ${\tilde{N}}>h$ (red and purple solid lines in the figure), the propagation speed is computed for $t>3$, as the population cannot be detected at any time $t<3$ (Color figure online)

An example of the propagation speed is shown in Fig. 2a, where we compare *c*(*t*) obtained from (13)–(14) with the propagation speed obtained by direct computation for the linear growth function (10); see (Deeley and Petrovskaya 2022) for further details of the numerical procedure. It can be seen from the figure that the result of the direct computation is in very good agreement with the analytical result, hence the accuracy of our computational method being confirmed. We then compute the propagation speed for the growth function (9), as the analytical solution is not available in the latter case. The propagation speed obtained for the function (9) very slightly differs from *c*(*t*) obtained for the linear growth (10), demonstrating that the linear growth is a good approximation of the growth at small densities in a transient regime before the establishment of a monotone travelling wave.

One result that can be seen from Fig. 2a is that the invasive species decelerates before the propagation speed converges to its asymptotic value given by (15). However, the deceleration regime is not common and depends on the choice of the detection threshold ${\tilde{N}}$. Several examples of the propagation speed are presented in Fig. 2b where the value of ${\tilde{N}}$ is varied. The propagation regime switches from a decelerating wave to an accelerating wave as the threshold density increases, and then the function *c*(*t*) becomes non-monotone before going back to a decelerating wave regime with the further increase of $\tilde{N}$. We also note that for ${\tilde{N}}>h$ detection of the high density ${\tilde{N}}$ becomes only possible at times $t>3$, and we cannot compute the propagation speed at shorter times; see Fig. 2b.

**Table 1:** Comparison of the propagation speed *c*(*t*) (13)–(14) and the asymptotic speed $c^*$ (15)

*t*	1	6	11	16	21	26
*c*(*t*)	0.0726	0.1291	0.1321	0.1331	0.1337	0.1340
$\epsilon _{rel}$	0.4637	0.0461	0.0242	0.0165	0.0125	0.0101

The relative rate of propagation $\epsilon _{rel}$ is given by (17) for the threshold density $\tilde{N} = 0.5h$, $h=0.1$ (cf. the propagation speed in Fig. 2b). The other parameters are $\Phi = 0.01$, $A=2.5$, and $\sigma =0.1$

Let us now investigate the question of how fast the propagation speed (14) will achieve its asymptotic value (15). One example of the convergence of the propagation speed is shown in Table 1 where we compute a relative difference between *c*(*t*) and $c^*$,

17 $$\begin{aligned} \displaystyle {\epsilon _{rel}=\frac{|c(t)-c^*|}{c^*}}. \end{aligned}$$

The results in the table demonstrate that, contrary to the visual representation in Fig. 2b, the propagation speed does not approach the asymptotic speed $c^*$ with reasonably good accuracy over the range of time presented there: we have the minimum value $\epsilon _{rel} \approx 10^{-2}$ for $t=26$ (cf. *c*(*t*) given by the orange solid line for $\tilde{N} = 0.5h$ in Fig. 2b, where visual analysis of the graph may result in a wrong conclusion of fast convergence). Hence, we introduce the transition time $t_{tran}$ as the time taken to converge to the constant propagation speed $c^*$ given by (15) with certain accuracy $\epsilon$. We have

18 $$\begin{aligned} \mid c(t, {\tilde{N}}) - {c}^* \mid < \epsilon , \quad \forall t \ge t_{tran}, \end{aligned}$$

where the accuracy $\epsilon$ can be thought of in terms of the asymptotic speed, $\epsilon = k{c}^*$ with $k \ll 1$. The transition time is then defined in computation as the minimum time for which the condition (18) holds.

**Fig. 3:**
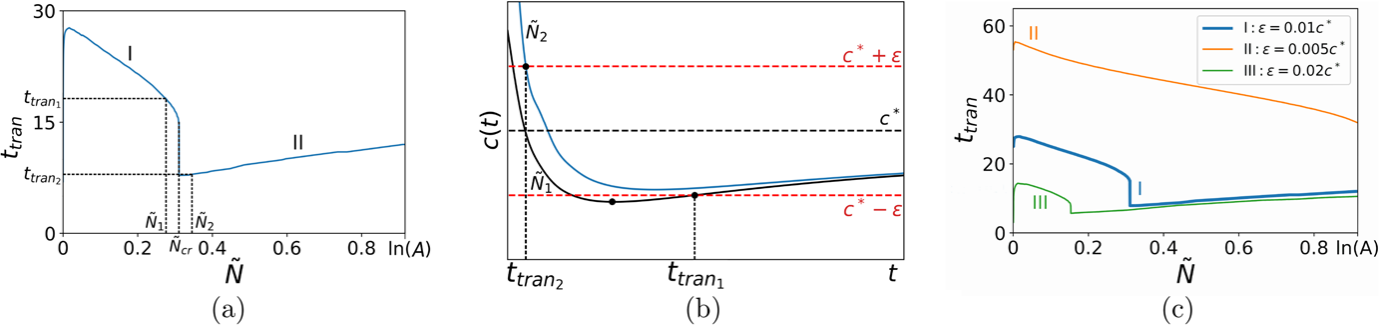
**a** The transition time $t_{tran}$ given by (18) against the detection threshold ${\tilde{N}}$. The ‘baseline’ case: the transition time computed for the choice of the parameters $\Phi = 0.01$, $A=2.5$, $\epsilon = 0.01c^*$, $h=0.1$, and $\sigma =0.1$. The transition time shows a non-monotone behaviour as the detection threshold increases and has a jump at ${\tilde{N}} = {\tilde{N}}_{cr}$. **b** The propagation speed computed for $\tilde{N}_1=2.75h \lessapprox {\tilde{N}}_{cr}$ and ${\tilde{N}}_2=3.50h \gtrapprox {\tilde{N}}_{cr}$ (see **a**). The turning point in the graph *c*(*t*) is outside the strip $[c^*-\epsilon , c^*+\epsilon ]$ when ${\tilde{N}} = {\tilde{N}}_1$ resulting in a significantly larger transition time (see further explanation in the text). **c** Various choices of the accuracy $\epsilon$ in the definition (18): the ‘baseline’ case, $\epsilon = 0.01{c^*}$ (blue solid line), the high accuracy, $\epsilon = 0.005{c^*}$ (orange solid line), the low accuracy, $\epsilon = 0.02{c^*}$ (green solid line). The other parameters in **c** are the same as in **a** (Color figure online)

It is immediately clear from (18) that the transition time depends on the detection threshold density ${\tilde{N}}$. One example of the transition time as a function of the detection threshold is presented in Fig. 3a. It can be readily seen from the figure that the transition time against the detection threshold is non-monotone and has a jump at the threshold density $\tilde{N}=\tilde{N}_{cr}$. Consider the threshold density $\tilde{N}_1$, where ${\tilde{N}}_1$ is close to $\tilde{N}_{cr}$ and belongs to the branch *I* of the graph in Fig. 3a. The propagation speed *c*(*t*) computed with the threshold ${\tilde{N}}_1$ is shown in Fig. 3b where we also show a strip of width $2\epsilon$ centered at $c^*$ as required to define the transition time in (18). The curve *c*(*t*) goes inside the strip $D_\epsilon =[c^*-\epsilon , c^*+\epsilon ]$ as a decelerating wave, leaves it after some time, and, after having a turning point $\displaystyle {\frac{\textrm{d}c(t)}{\textrm{d}t}=0}$, begins converging to the asymptotic speed $c^*$ as an accelerating wave. The transition time $t_{tran_1}$ is defined according to (18) when the curve *c*(*t*) enters the domain $D_\epsilon$ again; see Fig. 3b.

Consider now the threshold density ${\tilde{N}}_2$, where ${\tilde{N}}_2$ is close to $\tilde{N}_{cr}$ and belongs to the branch *II* of the graph in Fig. 3a. The curve *c*(*t*) goes inside the strip $D_\epsilon$ and remains there as time progresses. The transition time $t_{tran_2}$ is therefore defined when the curve *c*(*t*) enters the domain $D_\epsilon$ as a decelerating wave; see Fig. 3b. The analysis of Fig. 3b reveals that the critical value of the threshold density $\tilde{N}_{cr}$ can be defined under the requirement that the following conditions hold at time $t_{cr}$:

19 $$\begin{aligned} c(t_{cr},\tilde{N}_{cr}) = c^*-\epsilon , \,\,\,\, \frac{\partial c(t_{cr}, \tilde{N}_{cr})}{\partial t}=0, \end{aligned}$$

where the propagation speed $$c(t, \tilde{N})$$ is given by (14). Once the critical threshold density $$\tilde{N}_{cr}$$ and the time $$t_{cr}$$ have been found from (19), the magnitude *J* of a jump in the transition time is $$ J = t_{cr}-t_0, $$ where the time $$t_0$$ is defined from the condition

20 $$\begin{aligned} c(t_0,\tilde{N}_{cr}) = c^*+\epsilon . \end{aligned}$$

It follows from the above consideration that the position $\tilde{N}_{cr}$ and the magnitude *J* of a jump in the transition time depend on the selected accuracy $\epsilon$. The transition time for various choice of $\epsilon$ is shown in Fig. 3c, where the curve *I* is the baseline case of Fig. 3a ($\epsilon =0.01c^*$). Applying a smaller value of $\epsilon$ in the problem (curve *II*, $\epsilon _1=0.005c^*=0.5\epsilon$) results in the absence of a jump: all curves *c*(*t*) generated for the range $\tilde{N} \in (0, \ln (A)]$ now have the turning point $\displaystyle {\frac{\textrm{d}c(t)}{\textrm{d}t}=0}$ outside the strip $[c^*-0.5\epsilon , c^*+0.5\epsilon ]$ and the entire curve *II* in Fig. 3c is topologically similar to the branch *I* of the baseline case in Fig. 3a. The critical accuracy $\epsilon _{cr}$, such that the choice of any $\epsilon <\epsilon _{cr}$ in (18) results in a continuous curve $t_{tran}(\tilde{N})$, can be defined from the requirement $\tilde{N}_{cr}=\ln (A)$ combined with the conditions (19), i.e., we have

21 $$\begin{aligned} c(t_{cr},\ln (A)) = c^*-\epsilon _{cr}, \,\, \,\, \frac{\textrm{d}c(t_{cr}, \ln (A))}{\textrm{d}t}=0. \end{aligned}$$

We also note that employing a lower accuracy (curve *III*, $\epsilon _2=0.2c^*=2\epsilon$) results in a smaller jump: the curve $c(t, \tilde{N}_{cr})$ considered in the baseline case now has the turning point inside the strip $[c^*-2\epsilon , c^*+2\epsilon ]$ (cf. Fig. 3b), and a new value of $\tilde{N}_{cr}$ found from (19) will correspond to a smaller time $t_{cr}$.

**Fig. 4:**
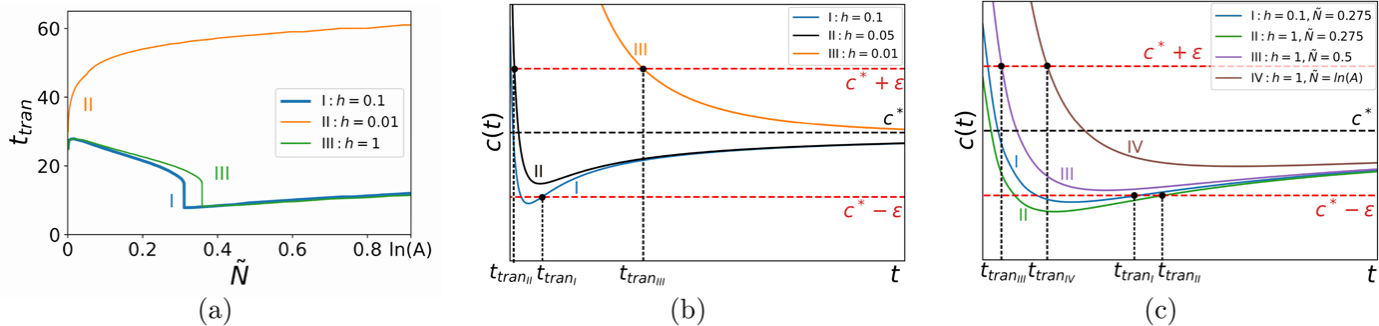
**a** The transition time $t_{tran}({\tilde{N}})$ for various choices of the height *h* of the initial population density: the ‘baseline’ case, $h=0.1$ (blue solid line), the low density, $h=0.01$ (orange solid line), and the high density, $h = 1$ (green solid line). The other parameters in this figure are the same as in Fig. 3a. **b** The behaviour of the propagation speed *c*(*t*) when the height *h* of the initial distribution decreases. The propagation speed *c*(*t*) in the baseline case of **a** shown for the threshold density $\tilde{N}_1=2.75h\lessapprox \tilde{N}_{cr}$ (curve *I*). Decreasing *h* moves the turning point inside the domain $D_\epsilon =[c^*-\epsilon , c^*+\epsilon ]$ (curve *II*). Further decrease in *h* makes the transition time longer (curve *III*). **c** The behaviour of the propagation speed *c*(*t*) when the height *h* of the initial distribution increases. The propagation speed *c*(*t*) in the baseline case of Fig. 3a shown for the threshold density $\tilde{N}_1=2.75h\lessapprox \tilde{N}_{cr}$ (curve *I*). Increasing *h* for the same threshold density $\tilde{N}_1$ moves the turning point farther from the domain $D_\epsilon$ (curve *II*) and the threshold density should be increased to place the turning point inside the strip $D_\epsilon$ (curve *III*). Further increase in $\tilde{N}$ keeps the propagation speed inside the domain $D_\epsilon$ yet increases the transition time (curve *IV*) (Color figure online)

Similar analysis based on the consideration of the propagation speed can be applied if we want to investigate how the transition time depends on the other parameters in (18). As one example of our approach, below we discuss the case of varying the height of the initial population *h*. Although detailed investigation of the impact which the initial condition makes on propagation on the short-time scale is beyond the scope of our paper (cf. Petrovskii and Shigesada 2001), the approach we use here can then be readily extended when the transition time is computed for propagation at the presence of a road in the next section. Consider the baseline case $t_{tran}(\tilde{N})$ of Fig. 3a which is also shown as curve *I* in Fig. 4a. The propagation speed *c*(*t*) in the baseline case is shown in Fig. 4b for the threshold density $\tilde{N}_1\lessapprox \tilde{N}_{cr}$, where the transition time is $t_{tran_1}$ (see curve *I* in Fig. 4b). We note that the turning point $\displaystyle {\frac{\textrm{d}c(t, \tilde{N}_1)}{\textrm{d}t}=0}$ of the curve *I* is outside the domain $D_\epsilon$. Let us decrease *h* keeping the other parameters the same as in the baseline case. The turning point goes inside the strip $D_\epsilon$ and remains there when smaller values of *h* are considered for the same threshold density $\tilde{N}_1$; see curves *II* and *III* in Fig. 4b. Meanwhile, it can also be concluded from Fig. 4a, c that decreasing the threshold density $\tilde{N}$ moves the turning point of *c*(*t*) along the *t*-axis to the left.

We now consider the propagation speed in (13)–(14) as $c=c(t,\tilde{N},\Phi (h))$, where $\Phi (h)$ is defined by (16). We then assume that there is the critical value $h_{cr}$ along with the unknown critical value of $\tilde{N}_{cr}$ for which the graph $c(t,\tilde{N}_{cr},\Phi (h_{cr}))$ has the turning point at $t=0$. Hence, the critical height $h_{cr}$ eliminating a jump in the graph $t_{tran}(\tilde{N})$ can be evaluated from the following conditions

22 $$\begin{aligned} \lim _{t\rightarrow 0}c(t,\tilde{N}_{cr}) = c^*-\epsilon , \,\,\,\, \lim _{t\rightarrow 0} \frac{\partial c(t,\tilde{N}_{cr},\Phi (h_{cr}))}{\partial t}=0. \end{aligned}$$

Implementation of any $h<h_{cr}$ in computation of the transition time (18) results in a continuous curve $t_{tran}(\tilde{N})$ (see curve *II* in Fig. 4a plotted for $h=0.01$).

Increasing the height *h* shifts the position $\tilde{N}_{cr}$ of the jump along the $\tilde{N}$-axis; see the baseline case (curve *I* in Fig. 4a) plotted for $h=0.1$ and compare it to curve *III* plotted for $h=1$ in the figure. The propagation speed *c*(*t*) in the baseline case is now shown as curve *I* in Fig. 4c and it corresponds to the transition time $t_{tran_1}$. Increasing *h* for the same threshold density $\tilde{N}$ results in a longer transition time $t_{tran_2}>t_{tran_1}$, while the turning point remains outside the strip $D_\epsilon$; see curve *II* in Fig. 4c. For the given height $h=1$, we have to increase $\tilde{N}$ to move the turning point (19) to the domain $D_\epsilon$ (see curves *III* and *IV* in Fig. 4c) where it remains for any $\tilde{N} > \tilde{N_{cr}}$. Hence, the critical value $\tilde{N_{cr}}$ is still found from (19), but we have $\tilde{N_{cr}}(h=1) > \tilde{N_{cr}}(h=0.1)$, i.e., the position of a jump moves to the right.

Overall, the transition time changes significantly as the detection threshold varies, and jumps in the graph depending on the parameters of the problem suggest that one cannot rely upon the transition time to predict or compare the propagation of the invasive species at short times before it transitions to the stable long-term behaviour. It is also important to note here that the choice of a high detection threshold $\tilde{N}$ results in a much shorter transition time in comparison with the case when a low detection threshold is used (cf. branches *I* and *II* of the graph in Fig. 3a).

Along with the transition time taken for the propagation speed to converge to the constant asymptotic speed, another question arising is: does the propagation regime at the short-time scale have any lasting impact on the distance *d* the invasive species propagates over a given time *T* from the location where it has been first detected? The size of a site occupied by invasive species as time progresses is of interest to practitioners as this information is required to quantify an increase or decrease in the invasive species in the geographic region and compare invasion patterns (Lonsdale 1999; Walker et al. 2023).

Let the population density ${\tilde{N}}$ be detected at $x_0$ at time $t=0$ and the same population density be observed at the location $x_T$ at time $t=T$. Finding the distance between those points requires integration of the propagation speed,

$$\begin{aligned} d = \int \limits _{t=0}^{t=T} c(t)dt = x(T) - x(0). \end{aligned}$$

In the model with discrete time, the integral is replaced by the sum ${d = \sum \limits _{t=1}^{t=T} c(t) \delta t}$, where *c* is the propagation speed given by (14) and $t \in {\mathbb {N}}$. Substituting *c* from (14), rearranging terms and taking $\delta t=1$ gives

23 $$\begin{aligned} d= & {} \sum _{t=1}^{T} (x_t({\tilde{N}}) - x_{t-1}({\tilde{N}})) = x_T(\tilde{N}) - x_0({\tilde{N}}) \nonumber \\= & {} \displaystyle \sqrt{2(\sigma ^2_0+T\sigma ^2) \ln {\left( \frac{\Phi A^T}{\tilde{N} \sqrt{2\pi (\sigma ^2_0+T\sigma ^2)}}\right) }} -\sqrt{{2\sigma _0^2} \ln {\left( \frac{\Phi }{\tilde{N} \sqrt{2\pi \sigma _0^2}}\right) }}. \end{aligned}$$

**Fig. 5:**
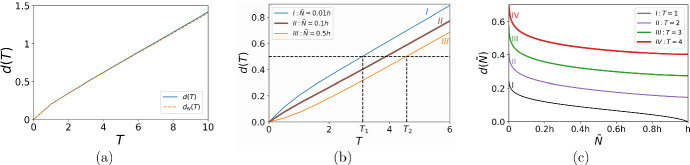
The distance *d* covered by the invasive species over the time *T* from the point $x_0$ where it has been detected at time $t=0$. **a** The comparison between the distance *d*(*T*) computed from the equation (23) (blue solid line) and the distance $d_N(T)$ obtained by the direct numerical simulation with the growth function (9) (orange dashed line). The parameters are $\Phi = 0.01$, $A=2.5$, $h=0.1$, $\sigma =0.1$, and $\tilde{N} = 0.01h$. **b** The distance (23) for various values of the detection threshold: $\tilde{N}=0.01h$ (blue solid line), $\tilde{N}=0.1h$ (brown solid line), and ${\tilde{N}}=0.5h$ (orange solid line). The time *T* required to cover a selected distance *d* depends on the detection threshold $\tilde{N}$: the given distance *d* (black dashed line) is covered faster ($T_1 <T_2$) when a lower detection threshold is selected. **c** The distance $d(\tilde{N})$ for $\tilde{N}\in (0, h]$ calculated for $T=1$ (black solid line), $T=2$ (purple solid line), $T=3$ (green solid line), and $T=4$ (red solid line). The other parameters are the same as in **a** (Color figure online)

The distance *d* the invasive species goes over a given time *T* from the point $x_0$ is shown in Fig. 5. The comparison between the distance computed from the equation (23) and the direct numerical simulation with the growth function (9) is presented in Fig. 5a, where the results are in very good agreement at the short times; cf. Fig. 2a. The distance covered by the invasive species over a given time *T* is shown in Fig. 5b. It can be seen from the figure that, given the distance *d* (a horizontal dashed line), the time *T* required to cover that distance depends on the detection threshold $\tilde{N}$, i.e., the time $T_1$ corresponding to the choice of a lower detection threshold is $T_1 <T_2$, where the time $T_2$ corresponds to the choice of a higher detection threshold.

Since the distance *d*(*T*) depends on the threshold density $\tilde{N}$, we show the graphs $d({\tilde{N}})$ in Fig. 5c for selected times *T*. We note from Fig. 5c that the distance $d({\tilde{N}})$ given by (23) is a monotone decreasing function of the threshold density ${\tilde{N}}$. In other words, the graph *d*(*T*) obtained for ${\tilde{N}}=0.01h$ (i.e., the smallest value of the threshold density used) is the upper bound for the other graphs in Fig. 5b, and this result confirms the importance of selecting the detection threshold density as small as possible when an evaluation of the size of a spatial domain invaded over a given period of time is made.

The spread rate *c*(*t*) can be non-monotone at the short-time scale as shown in Fig. 2b. Meanwhile, the results of Fig. 5 suggest that the distance *d*(*T*) is a monotone function of time and the threshold density ${\tilde{N}}$ alike. Hence, it is convenient to introduce $\bar{c}_T (\tilde{N})$, the average propagation speed over time *T*, in the problem. We have

$$\begin{aligned} \bar{c}_T (\tilde{N}) = \frac{1}{T}\int \limits _{t=0}^{t=T} c(t)dt = \frac{d(T)}{T}, \end{aligned}$$

and the average propagation speed remains a monotone function if *d*(*T*) is monotone. Taking into account the definition (23) of the distance *d*(*T*) in the model with discrete time gives

24 $$\begin{aligned} \bar{c}_T (\tilde{N}) = {\displaystyle \frac{1}{T} \left( \sqrt{2(\sigma ^2_0+T\sigma ^2) \ln {\left( \frac{\Phi A^T}{\tilde{N} \sqrt{2\pi (\sigma ^2_0+T\sigma ^2)}}\right) }} -\sqrt{{2\sigma _0^2}\ln {\left( \frac{\Phi }{\tilde{N} \sqrt{2\pi \sigma _0^2}}\right) }}\right) }. \nonumber \\ \end{aligned}$$

**Fig. 6:**
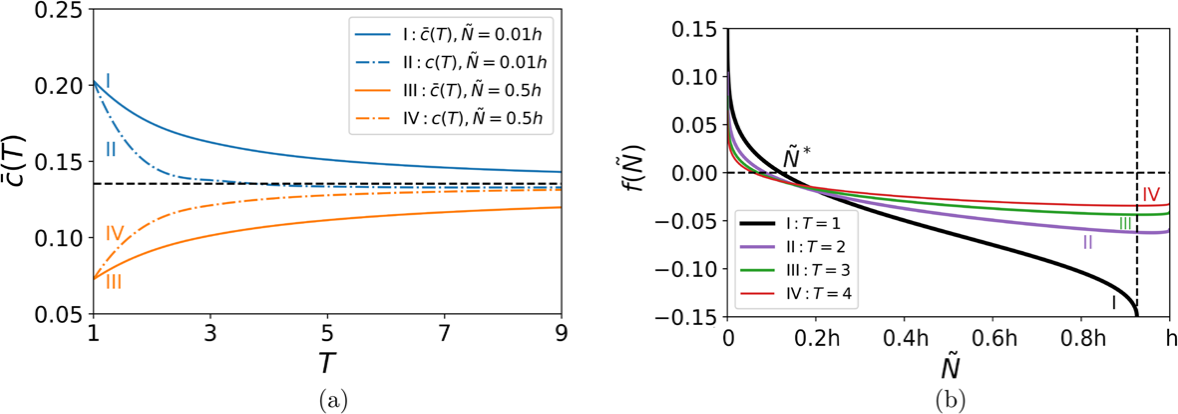
**a** The average propagation speed $\bar{c}_T$ (solid lines) is shown along with the instantaneous propagation speed *c*(*T*) given by (14) for $t=T$ (dashed lines). The propagation speeds are obtained for $\tilde{N} = 0.01h$ (blue solid and dashed lines) and $\tilde{N} = 0.5h$ (orange solid and dashed lines). Both average and instantaneous propagation speeds converge to the asymptotic propagation speed (15) (black dashed line in the figure), the average speed converging either as a decelerating wave or an accelerating wave for the entire range of time. The instantaneous speed may present a deceleration–cceleration regime (blue dashed line). **b** The function $f(\tilde{N})$ given by (25) is computed for $T=1$ (black solid line), $T=2$ (purple solid line), $T=3$ (green solid line), and $T=4$ (red solid line) when $\tilde{N}\in (0, h]$. The vertical dashed line represents the maximum value of the density threshold $\tilde{N}$ for which (25) can be determined at $T=1$. The other parameters in Fig. 6 are the same as in Fig. 5a. The equation $f(\tilde{N})=0$ has a single root at every time *T* where the largest root $\tilde{N^*}$ corresponds to $T=1$ (Color figure online)

The average propagation speed $\bar{c}_T$ along with the instantaneous propagation speed *c*(*T*) is shown in Fig. 6a for the detection threshold $\tilde{N}=0.01h$ and $\tilde{N}=0.5h$ (cf. Fig. 5b). For the low threshold $\tilde{N}=0.01h$, the average speed $\bar{c}_T (\tilde{N})$ represents a deceleration regime (unlike the instantaneous speed *c*(*t*) where the deceleration-acceleration regime appears), while $\bar{c}_T(\tilde{N})$ computed at the high threshold density $\tilde{N}=0.5h$ represents an accelerating wave over the entire range of time.

Consider now the function $f(\tilde{N})$ defined as

25 $$\begin{aligned} f(\tilde{N})=\bar{c}_T(\tilde{N})-c^*, \end{aligned}$$

where the average propagation speed is given by (24) and the asymptotic speed $c^*$ is given by (15). The graph of $f(\tilde{N})$ is presented in Fig. 6b for the same parameter values as in Fig. 5c. It can be seen from the graph that the equation $f(\tilde{N})=0$ has a single root at every time *T* where the largest root $\tilde{N^*}$ corresponds to $T=1$. If the detection threshold is chosen as $\tilde{N} > \tilde{N^*}$, then $f(\tilde{N})<0$ at times $T=1,2,3,4$. Hence, the average propagation speed is $\bar{c}_T(\tilde{N})<c^*$ and the observer will see an accelerating invasion wave (24) at the beginning of invasion (*cf*. curves $III-IV$ in Fig. 6a). We have already mentioned that our model is developed under the assumption of the unknown magnitude of the initial distribution, i.e., we assume that it is not possible to say whether the detection threshold $\tilde{N}$ selected for monitoring is sufficiently small. Meanwhile, the above consideration suggests an approach to evaluation of the magnitude of the threshold density $\tilde{N}$: observing an accelerating wave (24) at the beginning of invasion may indicate that the detection threshold used in the monitoring protocol is too high, $\tilde{N} > \tilde{N^*}$.

Once short-time dynamics have been studied in the homogeneous landscape, the next question to investigate is whether these dynamics become even more complex in the heterogeneous environment. In the next section, the heterogeneous landscape through the introduction of a road will be considered, where we will demonstrate how the propagation of the invasive species at short times is changed by the presence of heterogeneity in the model.

## Invasion at the Short-Time Scale in the Presence of a Road

The aim of this section is to investigate the impact of a ‘road’ in the model (4)–(8) on the propagation of invasive plants at the short-time scale. Let the road sub-domain $\Omega _R$ of width $\delta$ be defined by $\Omega _R=[b, b+\delta ]$, $\delta > 0$. As a travelling wave propagates behind the road, a certain amount of the population density is brought in front of the road at each time step. While that amount evolves over time into the same travelling wave as behind the road, we are interested in the transient regime before a travelling wave is established and propagates at a constant speed in front of the road.

**Fig. 7:**
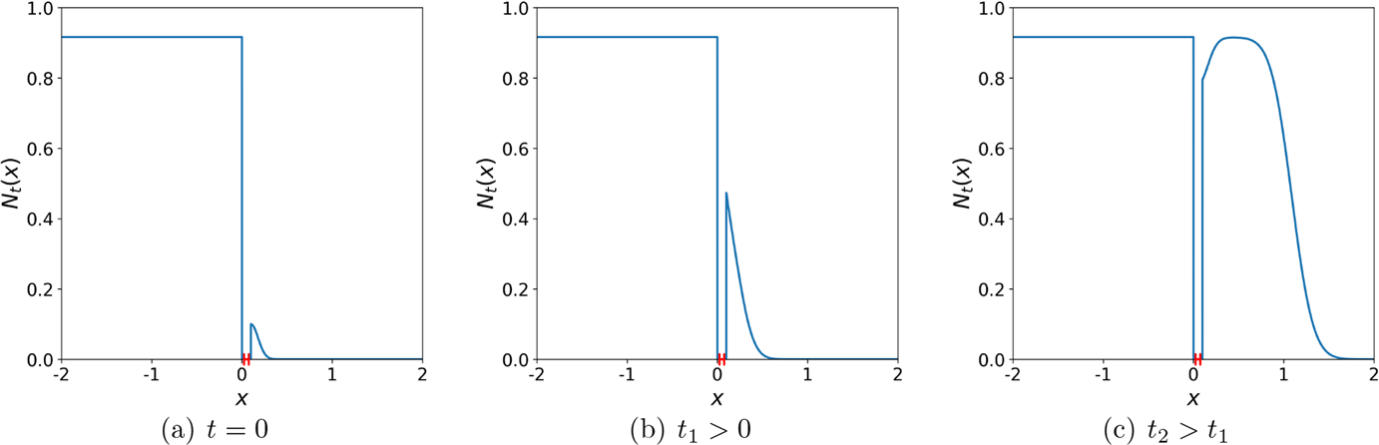
The propagation of the invasive species in the heterogeneous environment given by the model (4)–(8). The left edge of the road is positioned at $b=0$. The parameters used in computation are $\Phi = 0.01$, $h =0.1$, $\sigma = 0.1$, $\Psi \approx 0.016$, and $A=2.5$. **a** The initial population density in front of the road is considered as in (27). A travelling wave has already been established behind the road. **b** After some time $t_1>0$ the population density in front of the road grows and spreads further into the spatial domain, **c** At a later time $t_2>t_1$ the population density in front of the road continues to grow and spread into the domain. The same propagating wave as behind the road will be formed in front of the road as time progresses

The spatio-temporal dynamics in the transient regime is shown in Fig. 7, where we assume that a travelling wave behind the road has already been established, i.e., we have

26 $$\begin{aligned} N_t(x) = \ln {(A)}, \text {for } x\le b. \end{aligned}$$

Let the invasive species be first detected in front of the road at the time $t^*$. We then re-define the time variable as $\tilde{t}=t-t^*$ and omit the notation ${\tilde{t}}$ for the sake of convenience, using *t* instead. We assume that the population density behind the road is given by (26) for all times $t\ge 0$.

For the purpose of our study, we approximate the initial population density $N_0(x)$ in front of the road by a half-normal distribution, i.e., we consider the population density at $t=0$ as (cf. Fig. 7)

27 $$\begin{aligned} N_0(x) = \Phi \frac{\sqrt{2}}{\sqrt{\pi \sigma ^2_0}} \exp {\left( -\frac{(x-(b+\delta ))^2}{2\sigma ^2_0}\right) }, \text {for } x\ge b+\delta . \end{aligned}$$

The initial condition (27) in front of the road requires definition of the unknown dispersal parameter $\sigma _0$. Hence, we first specify the initial total population density in front of the road $\Phi$ and the height *h* of the population density at the edge of the road $x=b+\delta$ to be the same as in the initial condition (11). This is required to make the initial condition (27) compatible with the initial condition in the no road case. We note that the height *h* of the initial distribution in the road case is not taken from (16) as it now is provided by (27). Given the height *h* and the total population density $\Phi$ of the initial distribution (27), the dispersal parameter $\sigma _0$ is computed as

$$\begin{aligned} \sigma _0 = \frac{\Phi \sqrt{2}}{h \sqrt{\pi }}. \end{aligned}$$

The model then proceeds as follows. The initial condition (27) is transformed into a normal distribution

$$\begin{aligned} N^{normal}_0(x) = \Phi \frac{1}{\sqrt{2\pi \sigma ^2_0}} \exp {\left( -\frac{(x-(b+\delta ))^2}{2\sigma ^2_0}\right) }. \end{aligned}$$

The linear growth function (10) is now applied and the population density is dispersed at the time $t=1/2$, using the Gaussian dispersal kernel (3) with dispersal variable $\sigma$, to obtain

$$\begin{aligned} N^{normal}_{\frac{1}{2}}(x) = \Phi A \frac{1}{\sqrt{2\pi (\sigma ^2_0+\sigma ^2)}} \exp {\left( -\frac{(x-(b+\delta ))^2}{2(\sigma ^2_0+\sigma ^2)}\right) }. \end{aligned}$$

After the dispersal, the population density is transformed back into the form of a half-normal distribution,

$$\begin{aligned} N_{\frac{1}{2}}(x) = (\Phi A) \frac{\sqrt{2}}{\sqrt{\pi (\sigma ^2_0+\sigma ^2)}} \exp {\left( -\frac{(x-(b+\delta ))^2}{2(\sigma ^2_0+\sigma ^2)}\right) }, \text {for } x\ge b+\delta . \end{aligned}$$

Finally, the amount of the population density $\Psi =\Psi (\delta , \sigma )$ brought over from behind the road at the time $t=1$ is added to the population density in front of the road

28 $$\begin{aligned} N_1(x) = (\Phi A+\Psi ) \frac{\sqrt{2}}{\sqrt{\pi (\sigma ^2_0+\sigma ^2)}} \exp {\left( -\frac{(x-(b+\delta ))^2}{2(\sigma ^2_0+\sigma ^2)}\right) }, \text {for } x\ge b+\delta . \end{aligned}$$

The computation of the quantity $\Psi$ is provided in Appendix A. It is important to emphasise here that the amount of the population density brought over from behind the road depends on the road width $\delta$ and the dispersal strength $\sigma$, yet it does not depend on time as the population density behind the road is given by (26). Once the population density $\Psi$ has been computed for $t=1$, it can be further used for any $t>1$.

The expression (28) gives the population density at the time $t=1$. To calculate the population density at any time $t>0$, the above procedure is implemented to the population density at time $t-1$. Hence, an exact form for the population density in front of the road for any $t>0$ is given by

29 $$\begin{aligned} N_t(x) = (\Phi A ^ t + \Psi \sum _{i=0}^{t-1} A^i) \frac{\sqrt{2}}{\sqrt{\pi (\sigma ^2_0+t\sigma ^2)}} \exp {\left( -\frac{(x-(b+\delta ))^2}{2(\sigma ^2_0+t\sigma ^2)}\right) }, \text {for } x\ge b+\delta . \nonumber \\ \end{aligned}$$

Now that we have an analytical expression (29) for the population density, we can define a spatial location $x_t({\tilde{N}})>b+\delta$ in front of the road where the population density $\tilde{N}$ is detected at time *t*. For $t=0$, we solve $N_0(x_0)=\tilde{N}$ to find $x_0$, where the population density distribution $N_0$ is given by the initial condition (27). In the road case we have

$$\begin{aligned} x_0(\tilde{N}) = b+\delta + \sqrt{2\sigma ^2_0 \ln {\left( \frac{\Phi \sqrt{2}}{\tilde{N} \sqrt{\sigma ^2_0\pi }}\right) }}. \end{aligned}$$

Similarly, we solve $N_t(x_t)=\tilde{N}$ to find a spatial location $x_t$ for any $t>0$, where $N_t$ is given by (29),

30 $$\begin{aligned} x_t(\tilde{N},\Psi ) = b+\delta + \sqrt{2(\sigma ^2_0+t\sigma ^2) \ln {\left( \frac{(\Phi A ^ t + \Psi \sum _{i=0}^{t-1} A^i) \sqrt{2}}{\tilde{N}\sqrt{\pi (\sigma ^2_0+t\sigma ^2)}}\right) }}. \end{aligned}$$

The propagation speed $c=\displaystyle {\frac{\delta x}{\delta t}}$ is then calculated in the same way as in the homogeneous environment (14). Meanwhile, we note that, unlike the no road case, $x_t=x_t(\tilde{N}, \Psi )$ for any $t>0$, i.e., we have

31 $$\begin{aligned} c(t, \tilde{N}, \Psi ) = x_t (\tilde{N}, \Psi ) - x_{t-1}(\tilde{N}, \Psi ), \end{aligned}$$

where $x_t(\tilde{N}, \Psi )$ is defined from (30).

**Fig. 8:**
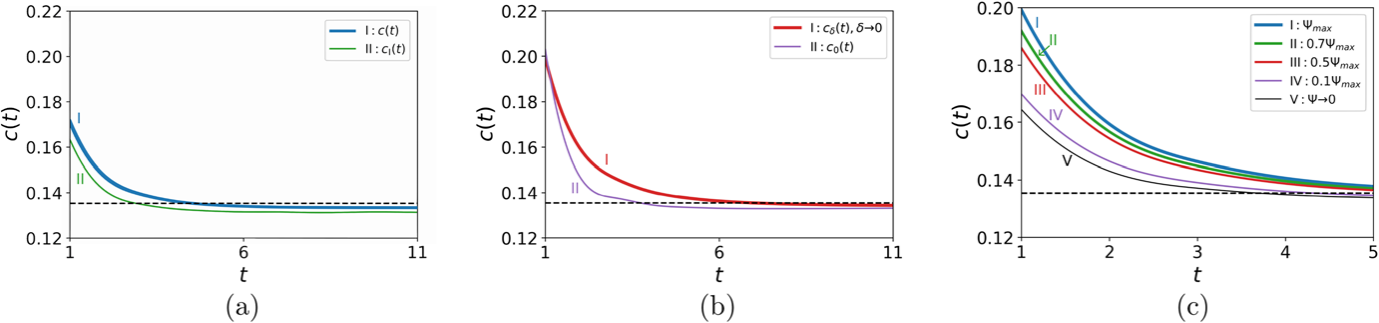
The propagation speed *c*(*t*) when the invasive plant begins spreading in front of the road. The asymptotic propagation speed $c^*$ given by (15) is shown as a black dashed line in the figure. **a** The comparison of the propagation speed *c*(*t*) obtained from (31) with $\Psi = 0.005$ (blue solid line) and the propagation speed $c_{I}(t)$ obtained by direct numerical computation with the growth function (9) (green solid line). **b** The comparison of the propagation speed $c_{\delta }(t)$ in the road model (31) for $\delta \rightarrow 0$ ($\Psi = \Psi _{max}$) (red solid line) and the propagation speed $c_{0}(t)$ in the no road model (14) (purple solid line). (c) The propagation speed (31) for $\Psi = \Psi _{max}\approx 0.037$ corresponding to the road width $\delta \rightarrow 0$ (blue solid line), $\Psi = 0.7\Psi _{max}$ (green solid line), $\Psi = 0.5\Psi _{max}$ (red solid line), $\Psi = 0.1\Psi _{max}$ (purple solid line), and $\Psi \rightarrow 0$ corresponding to the road width $\delta \rightarrow \infty$ (black solid line). The other parameters are $\Phi = 0.01$, $h=0.1$, $\tilde{N} = 0.01h$, $\sigma = 0.1$, and $A = 2.5$ (Color figure online)

The propagation speed computed by using (31) is shown in Fig. 8a where it is compared with the propagation speed obtained when the population density is computed by solving the model (4)–(8) with the growth function (9) numerically. It can be seen from the figure that, although approximation (27) of the population density in front of the road results in a slight difference between analytical and numerical results, the qualitative behaviour of the propagation speed is the same in both cases.

We then consider the extreme case of the road width $\delta \rightarrow 0$ in order to imitate the homogeneous environment. The investigation of this case is provided in the Appendix *A*, where the amount of the population density $\Psi _{max}=\Psi _{max}(\sigma )$ brought from behind an infinitely narrow road is defined by the formula (42). The comparison between the no road model and the road model (31) for $\Psi = \Psi _{max}$ is presented in Fig. 8b. It can be concluded from the graphs in the figure that the extreme case of an infinitely narrow road in the model (4)–(8) is not equivalent to the homogeneous environment when propagation at the short-time scale is considered. Indeed, it is assumed in our model that a travelling wave has already been formed behind the road and the wave behind the infinitely narrow road brings a constant amount $\Psi _{max}$ of the population density in front of the road at any time *t*. This is different from the no road case where we have the population growth from the initial condition (11) only, without adding a constant amount of the population density to (11) at each time step. Thus, adding the constant $\Psi _{max}$ to the population in front of the road results in a different propagation speed in the case of an infinitely narrow road. The difference, however, alleviates as time progresses and the initial population evolves into a travelling wave propagating at the constant asymptotic speed (15).

Another extreme case is given by a very wide road, i.e., the road width $\delta \rightarrow \infty$, where the additional population density brought from behind the road is negligible. The definition of the ‘negligible’ population density was investigated in our previous work (Deeley and Petrovskaya 2022) where the classification of ‘narrow’ and ‘wide’ roads was provided in the model. The infinitely wide road can then be considered by setting $\Psi = 0$ in the formula (29); see Appendix A for further explanation. The comparison between the road model (31) for $\Psi \rightarrow 0$ and $\Psi = \Psi _{max}$ is presented in Fig. 8c for a low detection threshold $\tilde{N}=0.01h$ (see curves *I* and *V*). Given the detection threshold, it is readily seen from the graphs that the invasive population spreads faster in the case of a very narrow road $\delta \rightarrow 0$ and making the road wider slows down a spread gradually (cf. curves *II*, *III*, and *IV* shown for interim values of $\Psi \in (0, \Psi _{max}]$).

**Fig. 9:**
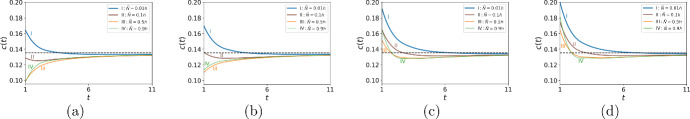
The propagation speed *c*(*t*) for various values of $\tilde{N}$, where the population density brought over the road is **a** $\Psi \rightarrow 0$ corresponding to $\delta \rightarrow \infty$, **b** $\Psi = 0.1\Psi _{max}\approx 0.0037$ corresponding to $\delta \approx 0.21$, **c** $\Psi = 0.7\Psi _{max}\approx 0.0259$ corresponding to $\delta \approx 0.05$, and **d** $\Psi = \Psi _{max}\approx 0.037$ corresponding to $\delta \rightarrow 0$. The threshold density values in **a**–**d** are $\tilde{N}=0.01h$ (blue solid lines), $\tilde{N}=0.1h$ (brown solid lines), $\tilde{N}=0.5h$ (orange solid lines), and $\tilde{N}=0.9h$ (green solid lines). The other parameters are $\Phi = 0.01$, $h=0.1$, $\sigma = 0.1$, and $A = 2.5$. The asymptotic propagation speed $c^*$ is shown as a black dashed line in the figure (Color figure online)

The examples of the propagation speed *c*(*t*) for various values of the detection threshold ${\tilde{N}}$ are shown in Fig. 9. We first vary ${\tilde{N}}$ when $\Psi \rightarrow 0$ (an infinitely wide road) where the results are provided in Fig. 9a. We note that the case of an infinitely wide road can be thought of as the ‘no road’ case in which the new (albeit similar) initial condition (27) is implemented instead of (11) and the results of Fig. 9a can be interpreted as the impact this change in the initial condition makes on propagation at the short-time scale. Thus, the underlying behaviour of the propagation speed against the detection threshold is similar to that in Fig. 2b.

The propagation speed is then computed for the same choice of ${\tilde{N}}$ but with a various amount of the population density $\Psi$ (Fig. 9b, c) increasing up to $\Psi =\Psi _{max}$ (an infinitely narrow road, Fig. 9d). It is seen from the figure that increasing $\Psi$ (i.e., decreasing the road width) makes the behaviour of the propagation speed more predictable as we now have $c(t)>c^*$ at small times, no matter what the value of ${\tilde{N}}$ is. The critical value of the population density $\Psi _0$ resulting in switching from an accelerating wave to a decelerating wave can be found from the requirement that a switch in the propagation regime already occurs at time $t=1$. Thus, we introduce the function $g(\Psi )$ as follows:

32 $$\begin{aligned} g(\Psi )=c(t=1,\tilde{N},\Psi )-c^*, \end{aligned}$$

where the propagation speed $c(t,\tilde{N},\Psi )$ is defined by (31).

**Fig. 10:**
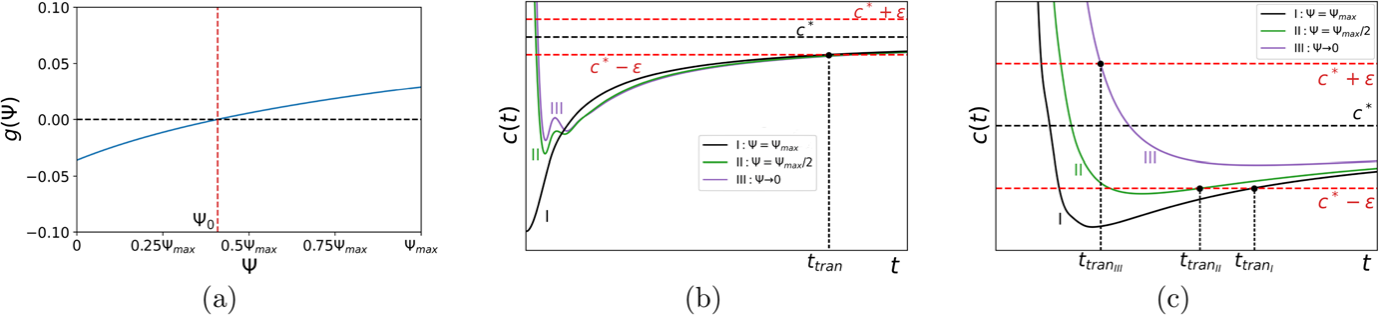
The propagation speed in the road case depends on the detection threshold density $\tilde{N}$ and the amount of the population density brought over the road $\Psi$. **a** Given the detection threshold $\tilde{N}=0.5h$, a switch from an accelerating wave to a decelerating wave occurs at $\Psi _0 = 0.408\Psi _{max}$ which corresponds to the road width $\delta = 0.106$ (see further explanation in the text). **b** The transition time is not significantly affected by the amount of the population density $\Psi$ and remains almost constant when it is measured at a low detection threshold $\tilde{N} = h$. **c** The transition time depends strongly on $\Psi$ when it is measured at a high detection threshold $\tilde{N} = 8h$. The other parameters in this figure are $h=0.1$, $\sigma = 0.1$, and $A = 2.5.$ (Color figure online)

An example of $g(\Psi )$ computed for $\tilde{N}=0.5h$ (see curves *III* in Fig. 9a–d) is shown in Fig. 10a where it can be seen that the function $g(\Psi )$ has a single root $\Psi _0$. For any $\Psi <\Psi _0$ the propagation speed measured at the given $\tilde{N}$ will present an accelerating wave at the beginning of invasion; see curves *III* in Fig. 9a, b. Conversely, for any $\Psi >\Psi _0$ a decelerating wave will be registered at the beginning of invasion (curves *III* in Fig. 9c, d).

**Fig. 11:**
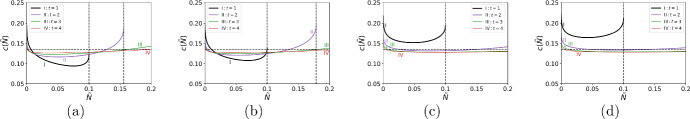
The propagation speed $c(\tilde{N})$ for various values of *t*, where the population density brought over the road is **a** $\Psi \rightarrow 0$ corresponding to $\delta \rightarrow \infty$, **b** $\Psi = 0.1\Psi _{max}\approx 0.0037$ corresponding to $\delta \approx 0.21$, **c** $\Psi = 0.7\Psi _{max}\approx 0.0259$ corresponding to $\delta \approx 0.05$, and **d** $\Psi = \Psi _{max}\approx 0.037$ corresponding to $\delta \rightarrow 0$. The time increments in **a**–**d** are $t=1$ (black solid lines), $t=2$ (purple solid lines), $t=3$ (green solid lines), and $t=4$ (red solid lines). The other parameters are $\Phi = 0.01$, $h=0.1$, $\sigma = 0.1$, and $A = 2.5$. The asymptotic propagation speed (15) is shown as a black horizontal dashed line in the figure and the vertical dashed lines represent the maximum value of the density threshold $\tilde{N}$ for which the propagation speed $c(\tilde{N})$ can be determined at the given time *t* (Color figure online)

Let us now calculate the propagation speed $c(\tilde{N})$ as a function of the detection threshold at various times. Again, we consider the extreme cases of $\Psi \rightarrow 0$ (infinitely wide road) and $\Psi =\Psi _{max}$ (infinitely narrow road) where the results are shown in Fig. 11a, d respectively. The results in the figure suggest that a rapid transition to the asymptotic regime occurs in the case of a narrow road in a spatial domain, while the propagation speed depends heavily on the detection threshold density as time progresses in the wide road environment.

**Fig. 12:**
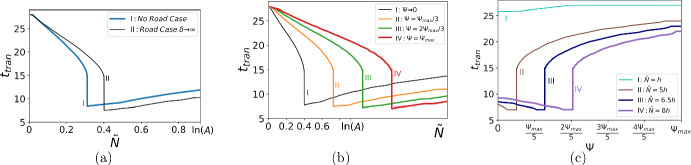
The transition time $t_{tran}$ at the presence of a road. The parameters used in computation are $\Phi = 0.01$, $A=2.5$, $\epsilon = 0.01c^*$, $h=0.1$, and $\sigma =0.1$ (cf. Fig. 3a). **a** The no road case (blue solid line) versus the road case as the road width $\delta \rightarrow \infty$, i.e., $\Psi \rightarrow 0$ (black solid line). **b** The transition time against the detection threshold density ${\tilde{N}}$ for various values of $\Psi$: the infinitely wide road $\Psi \rightarrow 0$ (black solid line), $\Psi = \Psi _{max} / 3$ (orange solid line), $\Psi = 2\Psi _{max}/3$ (green solid line), and the infinitely narrow road $\Psi = \Psi _{max}=0.037$ (red solid line). The range of ${\tilde{N}}$ is expanded beyond the upper bound ${\tilde{N}}=\ln (A)$ in the figure to show how the position of the jump changes as $\tilde{N}$ grows. **c** The transition time against the amount of the population density $\Psi$ for various values of the detection threshold density $\tilde{N}$: $\tilde{N} = h$ (turquoise solid line), $\tilde{N} = 5\,h$ (brown solid line), $\tilde{N} = 6.5h$ (blue solid line), and $\tilde{N} = 8h$ (purple solid line) (Color figure online)

Next, we compare the transition time (18) in the no road case and when the road width $\delta \rightarrow \infty$ in Fig. 12a. It is readily seen from the figure that the transition time is not the same in both cases as we have a different propagation speed at the short-time scale, as discussed earlier. The transition time depends now on the quantity $\Psi$ as well as the detection threshold ${\tilde{N}}$, and the transition time as a function of the threshold density ${\tilde{N}}$ is shown in Fig. 12b for various values of $\Psi$. It can be concluded from Fig. 12b that, with changing the amount of the population density brought over the road $\Psi$, a jump in the transition time persists for the entire range $\Psi \in (0, \Psi _{max}]$. The analysis proceeds as in the ‘no road’ case: we differ between the regimes where the turning point of the propagation speed *c*(*t*) is inside or outside the domain $D_\epsilon =[c^*-\epsilon , c^*+\epsilon ]$. Given $\Psi$, the position of the jump $\tilde{N}_{cr}$ can be found from the following conditions (see also the discussion in Sect. 3)

33 $$\begin{aligned} c(t_{cr},\tilde{N}_{cr}, \Psi ) = c^*-\epsilon , \,\,\,\, \frac{\partial c(t_{cr},\tilde{N}_{cr},\Psi )}{\partial t}=0, \end{aligned}$$

where the propagation speed $c(t, \tilde{N}, \Psi )$ is defined by (31).

We then analyse how the transition time changes when we vary the amount of the population density $\Psi$ for the given threshold density $\tilde{N}$; see Fig. 12c. Let us choose a low threshold density (curve *I* in Fig. 12c). Straightforward computation of the propagation speed reveals that the turning point $\displaystyle {\frac{\textrm{d}c(t)}{\textrm{d}t}=0}$ remains outside the domain $D_\epsilon$ for any $\Psi \in (0, \Psi _{max}]$; see Fig. 10b. Hence, there is no jump in the transition time as the propagation speed always converges to its asymptotic value $c^*$ as an accelerating wave. We also note that changing $\Psi$ does not change the transition time significantly when it is considered for a low threshold density $\tilde{N}$: we have $t_{tran} \approx const$ as can be seen in Fig. 10b (see also curve *I* in Fig. 12c).

Let us now consider a high threshold $\tilde{N}$ (curve *IV* in Fig. 12c) and compute the propagation speed for it. The graphs *c*(*t*) are shown for various $\Psi$ in Fig. 10c, where the curve *c*(*t*) has the turning point $\displaystyle {\frac{\textrm{d}c(t)}{\textrm{d}t}=0}$ outside the domain $D_\epsilon$ for large values of $\Psi$; see curve *I* in Fig. 10c. The transition time then becomes shorter as $\Psi$ decreases (curve *II*) and a further decrease in $\Psi$ results in the turning point moving inside the domain $D_\epsilon$ (curve *III*). Given $\tilde{N}$, the position of the jump $\Psi _{cr}$ is defined from the requirements

34 $$\begin{aligned} c(t_{cr},\tilde{N}, \Psi _{cr}) = c^*-\epsilon , \,\,\,\, \frac{\partial c(t_{cr},\tilde{N},\Psi _{cr})}{\partial t}=0. \end{aligned}$$

**Fig. 13:**
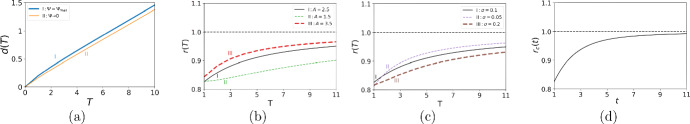
**a** The distance *d* covered by the invasive species over the time *T* from the point *x* in front of the road where it has been detected at time $t=0$. The amount of population density brought over from behind the road is given by $\Psi = \Psi _{max}$ (blue solid line) and $\Psi \rightarrow 0$ (orange solid line). The other variables are $\tilde{N}= 0.01h$, $\Phi = 0.01$, $A=2.5$, $h=0.1$, and $\sigma = 0.1$. **b** The relative size of the invaded domain *r*(*T*) given by (35) when the road width $\delta _1 \rightarrow \infty$ and $\delta _2 \rightarrow 0$. The baseline case (black solid line) has the same parameters as in **a**. Decreasing the growth factor *A* makes the ratio *r*(*T*) bigger (green dashed line, $A=1.5$), while increasing the growth alleviates the difference between a narrow and a wide road (red dashed line, $A=3.5$). **c** The relative size of the invaded domain *r*(*T*) depends also on the dispersal strength. The ratio *r*(*T*) shown for the baseline case of **a** (black solid line) becomes smaller as a result of weaker dispersal (purple dashed line, $\sigma =0.05$) and increases when the dispersal becomes stronger (brown dashed line, $\sigma =0.2$). **d** The ratio of propagation speeds (36) when the road width $\delta _1 \rightarrow \infty$ and $\delta _2 \rightarrow 0$ (Color figure online)

Finally, we investigate the distance *d* the invasive species goes over a given time *T* from the road edge $x=b+\delta$. The graphs *d*(*T*) are presented in Fig. 13a where we compare the case of a narrow road ($\delta \rightarrow 0$, $\Psi =\Psi _{max}$) and a wide road ($\delta \rightarrow \infty$, $\Psi \rightarrow 0$). The detection threshold density is selected as $\tilde{N}= 0.01h$ in line with the discussion of *d*(*T*) in Sect. 3. It is seen from the graphs that at any time *T*, the size of an invaded domain is smaller at the presence of a very wide road in comparison with the case of a very narrow road.

Let us introduce the invasion ratio *r*(*T*) as

35 $$\begin{aligned} r(T)=\frac{d(T, \delta _1)}{d(T, \delta _2)}, \end{aligned}$$

for two roads of width $\delta _1 \rightarrow \infty$ and $\delta _2 \rightarrow 0$, respectively. The function *r*(*T*) is shown as black solid line in Fig. 13b, where the relative size of the invaded region remains $r(T)<1$ for any $T>0$. Similarly, the propagation ratio $r_c(t)$ can be introduced as

36 $$\begin{aligned} r_c(t)=\frac{c(t, \delta _1)}{c(t, \delta _2)}, \end{aligned}$$

where $\delta _1$ corresponds to $\Psi \rightarrow 0$, $\delta _2$ corresponds to $\Psi =\Psi _{max}$ and we choose a low threshold density $\tilde{N}= 0.01h$ when the propagation speed is computed in (31). The propagation ratio $r_c(t)$ is shown in Fig. 13d, where it can be concluded from the figure that propagation is slower at the presence of a wide road (see also curves *I* and *V* in Fig. 8). The results obtained for *r*(*T*) and $r_c(t)$ agree with our previous conclusions: while the asymptotic propagation speed does not depend on the road width, propagation in a transient regime is slower in the presence of a wide road when a smaller amount of the population density is brought from behind the road at each time step. Hence, the construction of a wide road slows down invasion in the spatial domain in front of the road.

Let us also note that the two parameters defining the ratio *r*(*T*) are the growth factor *A* in (9) and the dispersal strength $\sigma$ in (3) as both of them make an impact on the amount of the population density $\Psi$ brought over the road into the domain in front of the road; see (42). A slower growth amplifies the effect of having a wide road in the domain (see curve *II* in Fig. 13b) as decreasing $\Psi _{max}$ makes the propagation speed slower (cf. Fig. 9). Conversely, increasing the growth factor *A* alleviates the impact of a wide road (curve *III* in Fig. 13b, where $\ln (A)=1.253$ slightly beyond the original range $0<\ln (A)<1$ is used to demonstrate the influence of the growth factor) as increasing $\Psi _{max}$ makes the propagation speed faster as shown in Fig. 9. The impact of the dispersal strength $\sigma$ on the ratio *r*(*T*) is presented in Fig. 13c. Since weaker dispersal slows down propagation, it makes a difference between a narrow road and a wide road smaller (cf. curves *I* and *II* in the figure). Conversely, stronger dispersion facilitates propagation and makes a bigger difference between a narrow road and a wide road (curve *III* in Fig. 13c). While further careful investigation of this topic is required, the results of Fig. 13 demonstrate that if the growth or propagation conditions of the invasive species change (e.g., because of a change in conditions of the environment) the same road may become less efficient in slowing down the invasion.

## Conclusions

In the present paper, we have investigated propagation of invasive plant species at the beginning of invasive spread in a homogeneous and heterogeneous environment. While the rate of spatial spread of the alien species has been a research topic of great interest for many years (Andow et al. 1990; Clark et al. 2001; Fisher 1937; Hastings 2005; Kot et al. 1996; Lewis and Pacala 2000), most studies have investigated the invasive spread at the long-time scale where the dispersal mode is a dominant factor defining either propagation at a constant speed or acceleration of a travelling wave (Baguette et al. 2012; Kot et al. 1996; Liu and Kot 2019). The early stage of the invasive spread has not received a lot of attention in the literature and it is often neglected by practitioners as it is assumed that the invasive species spreads out at the long-time scale regime from the very beginning of the invasion (Williamson 1996).

Invasive spread at the transition period can, however, be very different from propagation at longer times. In our work, the integro-difference framework has been employed to deal with a stage-structured population, and a short-distance dispersal mode has been considered. We have demonstrated that even in this relatively simple case when the propagation speed is asymptotically constant, strongly non-linear spatio-temporal dynamics appear at the transition regime. Furthermore, in our study transition to the asymptotic regime when a travelling wave invades a spatial domain at a constant rate has been observed over a time span of several generations (see Figs. 3 and 12). Long transition times suggest that assumptions about immediate establishment of the asymptotic regime of invasion need further investigation and the transition regime cannot be neglected.

The non-linear spatio-temporal dynamics manifests itself in various ways before the initial population density evolves into a travelling wave propagating at a constant speed. The propagation speed at the short-time scale is sensitive to the choice of the detection threshold density when the other parameters are fixed in the problem. It has been argued in the paper that the transition time to the asymptotic regime is also sensitive to the threshold density since the definition of the transition time is based on the propagation speed. One essential consequence of a non-linear behaviour of the propagation speed at the short-time scale is that a small change in the detection threshold can lead to an abrupt change (a jump) in the transition time and therefore the question of how to choose the detection threshold density at early stages of invasion requires further careful investigation.

The definition of the detection threshold density remains an open question in practical applications where very little information may be available about the initial density distribution. In our paper, we have considered an approach to estimating the detection threshold density based on the average propagation speed: observing an acceleration wave at the beginning of invasion may indicate that the detection threshold density used is too high (see Fig. 6). It is worth noting here that, while practitioners can see an accelerating wave at the beginning of invasion (Braithwaite 2010), a decelerating invasive wave corresponding to a low detection threshold is, to the best of our knowledge, not mentioned in the literature. Meanwhile, the results of our study suggest that a decelerating invasive wave registered for the smallest value of the detection threshold will also correspond to the longest distance covered by the invasive species over the given time (see Fig. 5b). Using an insufficiently small detection threshold density may result in a wrong conclusion about invasion, as the invasive species has already been spread in a larger spatial domain. While the requirement of having the lowest detection threshold density remains impractical in field observations (e.g., Walker et al. 2023), better understanding of how the choice of the detection threshold is related to observed properties of a spatial spread can help with the design of an efficient approach for preventing propagation of invasive species.

Spatial heterogeneity makes a further impact on the propagation speed of the invasive species during the transition from the initial distribution to the constant long-time behaviour. In our paper, the spatial heterogeneity was added in the form of a road in the landscape, where the road model is based on the previous work in Deeley and Petrovskaya (2022). In our study, the road is not considered as a ‘dispersal corridor’ but is modelled as a ‘hostile environment’ that results in an additional amount of the population density coming from the road to a spatial domain in front of the road at each time step, while the dispersal mode remains the same. It has been demonstrated in the paper that the constant amount of the population density brought over the road contributes to the non-linear behaviour of the propagation speed and the transition time at the short-time scale. This adds another layer of complexity to the problem, where, apart from the changes in the detection threshold density, small changes in the population brought to the spatial domain in front of the road can also lead to different values of the transition time.

Meanwhile, the amount of the population density brought over the road is defined by the road width, and that parameter alone can be used to control the propagation of the invasive species in the spatial domain. The distance the invasive species propagates over a given time from the location where it has been first detected in front of the road depends on the road width, and it has been argued in the paper that invasion slows down in the presence of a wide road. Furthermore, the above conclusion holds for propagation at the long-time scale: any spatial advance gained or lost in a transient regime will not alleviate when a travelling wave is established, as the asymptotic propagation speed does not depend on the road width. Thus, understanding the transient regime at the presence of a road is important, as it contributes to the overall propagation of the invasive species.

A 1-D layout presented in the paper can be thought of as a transect of the 2-D spatial domain where propagation is in the direction orthogonal to the road. It remains unclear, however, whether the results obtained in the 1-D case can be immediately extended to the more realistic 2-D landscape as in the latter case possible directional dispersal i.e., faster propagation of the invasive species along the road (van Putten et al. 2012) has to be taken into account. Directional dispersal may change the propagation regime at the short-time scale, and that issue will be investigated in future work.

Furthermore, incorporation of the directional dispersal into the problem may require redefinition of the dispersal kernel, i.e., employing long-distance dispersal in the direction along the road. While long-distance dispersal is indeed the primary dispersal type for many invasive plants (Kot et al. 1996; Nathan et al. 2011; Straigytė et al. 2015; Thuiller et al. 2006), its analysis remains a challenging issue, both from a mathematical and computational viewpoint. For example, our investigation in the present paper was essentially based on the existence of the constant asymptotic speed, e.g., see the definition of the transition time (18). Meanwhile, there is no constant asymptotic speed in problems with long-distance dispersal where the dispersal kernel is not exponentially bounded (Kot et al. 1996) and therefore the definitions used in the present study will require revision in future work.

It is also worth noting here that formation of an accelerating wave at the beginning of invasion observed by practitioners may occasionally result in confusing short-distance dispersal with an accelerating wave appearing when long-distance dispersal kernels are considered. For instance, it has been mentioned in Braithwaite (2010) that the spatial spread observed in their field studies had initial properties related to long-distance dispersal predominating and then properties related to short-distance dispersal predominating. Based on their observations, it has been concluded in Braithwaite (2010) that the complex spatial spread can be attributed to a possible mix of long distance and short-distance dispersal events. Meanwhile, our results demonstrate that a complex behaviour of the spatial spread, (e.g., an accelerating wave going to the asymptotic speed in Fig. 2 or a wave decelerating first, propagating nearly at the asymptotic speed for some time, accelerating and returning to the asymptotic speed at the end of the transition period in Fig. 3, may occur in the short-distance dispersal mode alone and is not related to long-distance dispersal. Hence, the issue of identifying long-distance dispersal events at the early stage of biological invasion requires further careful attention.

A more realistic problem formulation may also include a definition of the carrying capacity depending on the distance from the road as the growing conditions in the spatial domain close to the road may be different (Hansen and Clevenger 2005). Also, consideration of growth functions with the Allee effect remains another important stream of research. The mathematical theory behind the population growth damped by the Allee effect is different from the growth governed by (9) (e.g., see (Lewis et al. 2016)) and new transient regimes may arise when the Allee effect is introduced in the model. For example, it has been demonstrated in Deeley and Petrovskaya (2022) (see also Keitt et al. 2001) that the Allee effect is responsible for the formation of a so-called ‘beachhead’ regime where a small amount of the population density stands at the road edge. While the beachhead population does not propagate in front of the road, a small change in conditions of the environment can initiate its propagation resulting in invasion of the domain where the invasive species previously could not spread. Hence, the study of the initial phase of the beachhead propagation is important to prevent further spread of the invasive species, and it is considered as another topic of future work.

## Data Availability

Data sharing not applicable to this article as no datasets were generated or analysed during the current study.

## Appendix A

### Calculation of the Amount of the Population Density Brought Over the Road

Here we explain how to calculate the amount of the population density $\Psi =\Psi (\sigma , \delta )$, brought from behind the road of width $\delta$, to in front of the road at each time increment. In Deeley and Petrovskaya (2022), the population density was calculated in the heterogeneous landscape for the next time increment, given the initial population density condition,

37 $$\begin{aligned} N^*(x) = {\left\{ \begin{array}{ll} \ln {(A)}, &{} \text {for } x \in [a, b], \\ 0, &{} \text {otherwise}, \end{array}\right. } \end{aligned}$$

where *b* is the left hand edge of the road, and $a<b$. We want to calculate the amount of population density that spread from behind the road $x\in (-\infty , b]$, to in front of the road $x\in [b+\delta , \infty )$. Hence, in line with the initial condition behind the road (26), we choose $|b-a|$ to be sufficiently large to approximate the whole domain behind the road.

To calculate the total population density spread in front of the road from behind the road $\Psi (\delta , \sigma )$, with the initial condition (37), we need to solve

38 $$\begin{aligned} \Psi (\delta , \sigma ) = \ln {(A)} \int _{b+\delta }^{\infty } \phi (x, \delta ) \; dx, \end{aligned}$$

where $\phi (x, \delta )$ (the calculation of $\phi (x, \delta )$ can be found in Deeley and Petrovskaya (2022)) is given by

$$\begin{aligned}{} & {} \phi (x,\delta ) = \lambda _1(\nu _1(\lambda _2, \lambda _3, \lambda _4, \lambda _5, \lambda _6)-\nu _1(\lambda _2, \lambda _3, \lambda _4, \lambda _7, \lambda _6) \\{} & {} \quad -\nu _1(\lambda _8, \lambda _{9}, \lambda _{4}, \lambda _{10}, \lambda _{6}) + \nu _1(\lambda _8, \lambda _{9}, \lambda _{4}, \lambda _{11}, \lambda _{6})) + \frac{1}{2}(\nu _2), \end{aligned}$$

where,

39 $$\begin{aligned} \nu _1(\alpha _1, \alpha _2, \alpha _3, \alpha _4, \alpha _5)&= \exp {\left( \alpha _1+\alpha _2x+\alpha _3x^2\right) } {{\,\textrm{erf}\,}}{(\alpha _4 + \alpha _5x)}, \end{aligned}$$

40 $$\begin{aligned} \nu _2&= {{\,\textrm{erf}\,}}\left( \frac{b-x}{\sigma \sqrt{2}}\right) - {{\,\textrm{erf}\,}}\left( \frac{a-x}{\sigma \sqrt{2}}\right) , \end{aligned}$$

for $$\alpha _i \in \mathbb {R}$$, $$i=1,\ldots ,5$$, and

$$\begin{aligned}&\lambda _1 = \frac{1}{4} \frac{1}{\sqrt{d_1}} \frac{1}{\sqrt{2\sigma ^2}}, \quad \lambda _2 =\frac{c_1^2\sigma ^2-2\sqrt{2}bc_1\sigma +2c_2b^2}{4(1-c_2)\sigma ^2}, \quad \lambda _3 = \frac{\sqrt{2}c_1\sigma -2bc_2}{2(1-c_2)\sigma ^2}, \\&\lambda _4 = \frac{c_2}{2(1-c_2)\sigma ^2}, \quad \lambda _5 = \frac{2b+2\delta (1-c_2) -\sqrt{2}c_1\sigma }{4\sqrt{d_1}\sigma ^2}, \quad \lambda _6 = \frac{-1}{2\sigma ^2\sqrt{d_1}}, \\&\lambda _7 = \frac{2b-\sqrt{2}c_1\sigma }{4\sigma ^2\sqrt{d_1}}, \quad \lambda _8 = \frac{\sqrt{2}c_1\sigma -2ac_2}{2(1-c_2)\sigma ^2}, \quad \lambda _{9} = \frac{c_1^2\sigma ^2-2\sqrt{2}ac_1 \sigma + 2a^2c_2}{4\sigma ^2(1-c_2)}, \\&\lambda _{10} =\frac{2(1-c_2)(b+\delta )-\sqrt{2} \sigma c_1+2ac_2}{4\sigma ^2\sqrt{d_1}} \quad \lambda _{11} =\frac{2b(1-c_2) -\sqrt{2}c_1\sigma + 2ac_2}{4\sigma ^2 \sqrt{d_1}}. \end{aligned}$$

In order to calculate $\Psi$ from (38), one is required to integrate terms of the form $\nu _1$ (39). The substitution used previously in Deeley and Petrovskaya (2022), ${{\,\textrm{erf}\,}}{(x)} \approx 1 - \exp {(c_1 x + c_2 x^2})$, will be applied here again, where the coefficients $c_1$ and $c_2$ have been evaluated in Tsay et al. (2013) as $c_1 = -1.09599814703333$, and $c_2 = -0.75651138383854$. Using this approximation one can now calculate the integral of $\nu _1$ when $x\in [m,n]$. The two cases required to consider are as follows.

Case *I*: When $$\alpha _1+\alpha _2 x\ge 0$$,

$$\begin{aligned}&\int _{m}^{n} \nu _1\left( \alpha _1, \alpha _2, -\alpha _3, \alpha _4, \alpha _5\right) \; dx \\&\quad \approx \frac{\sqrt{\pi } \exp {\left( \frac{\alpha _2^2}{4\alpha _3} +\alpha _1\right) }}{2\sqrt{\alpha _3}} \left( {{\,\textrm{erf}\,}}{\left( \frac{\alpha _2 -2\alpha _3 m}{2\sqrt{\alpha _3}}\right) } -{{\,\textrm{erf}\,}}{\left( \frac{\alpha _2 -2\alpha _3 n}{2\sqrt{\alpha _3}}\right) }\right) \\&\qquad - \frac{\sqrt{\pi }\exp {\left( \frac{\beta _2^2}{4\beta _3} +\beta _1\right) }}{2\sqrt{\beta _3}}\left( {{\,\textrm{erf}\,}}{\left( \frac{\beta _2 -2\beta _3 m}{2\sqrt{\beta _3}}\right) } - {{\,\textrm{erf}\,}}{\left( \frac{\beta _2 -2\beta _3 n}{2\sqrt{\beta _3}}\right) }\right) \\&\quad =w_1\left( \alpha _1, \alpha _2, -\alpha _3, \alpha _4, \alpha _5 ; m,n\right) . \end{aligned}$$

Case *II*: When $\alpha _1+\alpha _2 x<0$,

$$\begin{aligned}&\int _{m}^{n} \nu _1\left( \alpha _1, \alpha _2, -\alpha _3, \alpha _4, \alpha _5\right) \; dx \\&\quad \approx \frac{\sqrt{\pi } \exp {\left( \frac{\beta _5^2}{4\beta _3} +\beta _4\right) }}{2\sqrt{\beta _3}} \left( {{\,\textrm{erf}\,}}{\left( \frac{\beta _5 -2\beta _3 m}{2\sqrt{\beta _3}}\right) } - {{\,\textrm{erf}\,}}{\left( \frac{\beta _5 - 2\beta _3 n}{2\sqrt{\beta _3}}\right) }\right) \\&\qquad - \frac{\sqrt{\pi }\exp {\left( \frac{\alpha _2^2}{4\alpha _3} +\alpha _1\right) }}{2\sqrt{\alpha _3}} \left( {{\,\textrm{erf}\,}}{\left( \frac{\alpha _2 -2\alpha _3 m}{2\sqrt{\alpha _3}}\right) } -{{\,\textrm{erf}\,}}{\left( \frac{\alpha _2 -2\alpha _3 n}{2\sqrt{\alpha _3}}\right) }\right) \\&\quad =w_2\left( \alpha _1, \alpha _2, -\alpha _3, \alpha _4, \alpha _5 ; m,n\right) , \end{aligned}$$

where $\beta _i$, $i=1,\ldots ,5$ are defined as

$$\begin{aligned}&\beta _1 = \alpha _4^2c_2 + \alpha _4c_1 + \alpha _1, \quad \beta _2=2\alpha _4\alpha _5c_2 + \alpha _5c_1 + \alpha _2, \quad \beta _3 = -\alpha _5^2c_2+\alpha _3, \\&\beta _4 = \alpha _4^2c_2 - \alpha _4c_1 + \alpha _1, \quad \beta _5 = 2\alpha _4\alpha _5c_2 - \alpha _5c_1 + \alpha _2. \end{aligned}$$

Next, one also has to calculate the integral of the form $\nu _2$ (40), when $x \in [b+\delta , \infty )$. The solution proceeds as follows,

$$\begin{aligned} \int _{b+\delta }^{\infty } \nu _2 \; dx&= b-a + \delta {{\,\textrm{erf}\,}}{\left( \frac{\delta }{\sqrt{2}\sigma }\right) } -\left( a-b-\delta \right) {{\,\textrm{erf}\,}}{\left( \frac{a-b-\delta }{\sqrt{2}\sigma }\right) }\\&\quad +\frac{\sqrt{2}\sigma }{\sqrt{\pi }} \left( \exp {\left( \frac{-\delta ^2}{2\sigma ^2}\right) } -\exp {\left( \frac{-\left( a-b-\delta \right) ^2}{2\sigma ^2}\right) }\right) = w_3. \end{aligned}$$

Finally, we define $M\left( \omega \right) = \max {\left( \omega , b+ \delta \right) }$, and the integral becomes

41 $$\begin{aligned}&\Psi \left( \delta , \sigma \right) \nonumber \\&\quad =\ln {\left( A\right) } \int _{b+\delta }^{\infty }\phi \left( x,\delta \right) \; dx \nonumber \\&\quad =\ln {\left( A\right) } \Bigg [\lambda _1 \Bigg (w_1\left( \lambda _2, \lambda _3, -\lambda _4, \lambda _5, \lambda _6 ; b+\delta , M\left( b+\delta -c_2\delta -\frac{1}{\sqrt{2}}c_1\sigma \right) \right) \nonumber \\&\qquad \qquad + w_2\left( \lambda _2, \lambda _3, -\lambda _4, \lambda _5, \lambda _6 ; M\left( b+\delta -c_2\delta -\frac{1}{\sqrt{2}}c_1\sigma \right) , \infty \right) \nonumber \\&\qquad \qquad - w_1\left( \lambda _2, \lambda _3, -\lambda _4, \lambda _7, \lambda _6 ; b+\delta , M \left( b-\frac{1}{\sqrt{2}}c_1\sigma \right) \right) \nonumber \\&\qquad \qquad - w_2\left( \lambda _2, \lambda _3, -\lambda _4, \lambda _7, \lambda _6 ; M\left( b-\frac{1}{\sqrt{2}}c_1\sigma \right) , \infty \right) \nonumber \\&\qquad \qquad - w_1\left( \lambda _8, \lambda _{9}, -\lambda _{4}, \lambda _{10}, \lambda _{6} ; b+\delta , M\left( \left( b+\delta \right) \left( 1-c_2\right) +ac_2 -\frac{1}{\sqrt{2}}c_1\sigma \right) \right) \nonumber \\&\qquad \qquad - w_2\left( \lambda _8, \lambda _{9}, -\lambda _{4}, \lambda _{10}, \lambda _{6} ; M\left( \left( b+\delta \right) \left( 1-c_2\right) +ac_2-\frac{1}{\sqrt{2}}c_1\sigma \right) , \infty \right) \nonumber \\&\qquad \qquad + w_1\left( \lambda _8, \lambda _{9}, -\lambda _{4}, \lambda _{11}, \lambda _{6} ; b+\delta , M\left( b\left( 1-c_2\right) +ac_2-\frac{1}{\sqrt{2}}c_1\sigma \right) \right) \nonumber \\&\qquad \qquad + w_2\left( \lambda _8, \lambda _{9}, -\lambda _{4}, \lambda _{11}, \lambda _{6} ; M\left( b\left( 1-c_2\right) +ac_2-\frac{1}{\sqrt{2}}c_1\sigma \right) , \infty \right) \Bigg ) + \frac{1}{2}w_3\Bigg ]. \end{aligned}$$

Below we calculate the amount of the population density $\Psi$ in the extreme cases of an infinitely wide and infinitely narrow road.

### Infinitely Wide Road

The first case we investigate is when the width of the road is $\delta \rightarrow \infty$. We note that the total amount of population density is

$$\begin{aligned} \int _{-\infty }^{\infty } \phi \left( x, \delta \right) \; dx = |b-a| \ln {\left( A\right) }:= C, \end{aligned}$$

where *C* is a constant for any given *a*. Thus the amount of the population density $\Psi \left( x, \delta \right)$ (41) in front of the road is

$$\begin{aligned} \Psi \left( x, \delta \right) = \int _{-\infty }^{\infty } \phi \left( x, \delta \right) \; dx - \int ^{X}_{-\infty } \phi \left( x, \delta \right) \; dx = C - \int ^{X}_{-\infty } \phi \left( x, \delta \right) \; dx, \end{aligned}$$

where $X = b+\delta$. For an infinitely wide road $\delta \rightarrow \infty$, the spatial coordinate is $X \rightarrow \infty$, and we have

$$\begin{aligned} \Psi \left( x, \delta \right)&= C - \lim _{X \rightarrow \infty } \int ^{X}_{-\infty } \phi \left( x, \delta \right) \; dx \\&= C-C = 0. \end{aligned}$$

Hence, the road acts as a ‘barrier’ preventing the invasive spread in the domain $x>b$, cf. (Deeley and Petrovskaya 2022).

### Infinitely Narrow Road

The second case we investigate is what happens when the width of the road is taken to be $\delta \rightarrow 0$. It has been shown in Deeley and Petrovskaya (2022) that $\phi \left( \delta \right)$ is a decreasing function of the road width $\delta$. Hence, the amount of the population density brought over the road $\Psi \left( \delta , \sigma \right)$ has a maximum value $\Psi _{max}$ at $\delta = 0$,

$$\begin{aligned} \Psi _{max}\left( \delta , \sigma \right) = \Psi \left( 0, \sigma \right) = \ln {\left( A\right) }\int _b^{\infty } \phi \left( x, 0\right) \;dx. \end{aligned}$$

Substituting $\phi \left( x, 0\right)$ and calculating the integral we arrive at

$$\begin{aligned} \Psi _{max}\left( \delta , \sigma \right) = \ln {\left( A\right) } \frac{1}{2}\left( \left( b-a\right) \left( {{\,\textrm{erf}\,}}{\left( \frac{a-b}{\sqrt{2}\sigma }\right) } + 1\right) + \frac{\sqrt{2}\sigma }{\sqrt{\pi }} \left( 1 - \exp {\left( \frac{-\left( a-b\right) ^2}{2\sigma ^2}\right) }\right) \right) . \end{aligned}$$

We now take $a\rightarrow -\infty$ to obtain

42 $$\begin{aligned} \Psi _{max}\left( \delta , \sigma \right) =\ln {\left( A\right) }\frac{\sigma }{\sqrt{2\pi }}. \end{aligned}$$
